# Designing an mHealth App to Encourage Uptake of Muscle-Strengthening Exercise in Older Adults: Co-Design Focus Group Study

**DOI:** 10.2196/87332

**Published:** 2026-03-12

**Authors:** Ethan CJ Berry, Nicholas F Sculthorpe, Ashley Warner, Nilihan EM Sanal-Hayes, Lawrence D Hayes

**Affiliations:** 1 Division of Sport, Exercise and Health University of the West of Scotland Glasgow, Scotland United Kingdom; 2 School of Health and Society University of Salford Manchester, England United Kingdom; 3 Lancaster Medical School Lancaster University Lancaster, England United Kingdom

**Keywords:** aged, co-design, mHealth, muscle-strengthening, older adults, telemedicine

## Abstract

**Background:**

Sarcopenia, the age-related decline in muscle mass and strength, poses a significant threat to functional independence in older adults. Despite strong evidence supporting resistance training as a preventive and therapeutic strategy, adherence to muscle-strengthening guidelines remains low. Mobile health (mHealth) technologies offer a promising avenue to bridge this gap; however, few apps are tailored to older adults or designed with their input.

**Objective:**

This study aimed to identify key features that a muscle-strengthening exercise app should include to enhance engagement and uptake among older adults. Secondary aims were to explore perceived barriers and facilitators to app use and to inform the development of an evidence-based, co-designed mHealth intervention.

**Methods:**

We used a qualitative co-design approach, involving 4 focus groups with 18 older adults (aged 60-83 years); each group comprised 3 to 6 older adults, stratified by experience with mHealth apps. Sessions were conducted online via Microsoft Teams and guided by a semistructured protocol informed by prior mHealth research and behavior change theory. Transcripts were analyzed using deductive thematic analysis, underpinned by the Technology Acceptance Model, focusing on perceived usefulness and perceived ease of use.

**Results:**

A total of 4 overarching themes and 10 subthemes were identified. Theme 1, mHealth as a tool for supporting health and well-being, highlighted participants’ recognition of digital tools in promoting activity and overcoming accessibility barriers. Theme 2, motivation and engagement through app features, revealed the importance of reminders, progress tracking, and feedback, although views on gamification were mixed. Theme 3, drawbacks of current mobile apps, captured concerns around complexity, poor usability, and lack of age-appropriate content, with skepticism regarding safety and evidence base. Theme 4, desired app elements and features, emphasized the need for customizable reminders, clear instructional videos, adaptable exercise options, and optional social features. Participants stressed the importance of simplicity, personalization, and relatable content to foster trust and sustained engagement.

**Conclusions:**

Older adults are receptive to mHealth interventions for muscle-strengthening when design is user centered and grounded in their lived experiences. This study provides a framework for future app development, highlighting the need for intuitive interfaces, personalized features, and credible educational content. By aligning design with Technology Acceptance Model constructs and co-design principles, mHealth apps can better support healthy aging and sarcopenia prevention. These findings offer actionable guidance for developers and researchers aiming to enhance digital health equity and effectiveness in older populations.

**Trial Registration:**

Open Science Framework 10.17605/OSF.IO/J64ER; https://osf.io/j64er/overview

## Introduction

### Background

Aging is a universal phenomenon, and in recent years, the global population has aged at an unprecedented rate [1]. Projections indicate that by 2050, 22% of the world’s population will be aged 65 years or older [1]. Aging brings several health challenges, including increased risks of dementia and cognitive impairment, elevated blood pressure, heart disease, and diabetes mellitus [2,3]. These health issues significantly impact the ability of older adults to perform daily activities. For example, cardiovascular issues experienced in older age can lead to fatigue and shortness of breath, making everyday tasks difficult and in turn setting off a cascade of inactivity [4]. Diabetes can lead to peripheral neuropathy, vision loss, and fatigue in older adults, in turn impacting balance, fine motor tasks, driving, reading, and cooking [5]. Consequently, health and well-being have become priority areas, as reflected in the United Nations Sustainable Development Goals, particularly the goal of ensuring healthy lives and well-being for all ages [6].

One key age-related health issue is sarcopenia, which is defined as the progressive deterioration of muscle mass and strength [7,8]. This phenomenon occurs with age as type II muscle fibres and satellite cells decline, which are crucial for muscle repair [9]. Furthermore, aging causes mitochondrial dysfunction, meaning energy production in the muscle is reduced and atrophy occurs [10]. Factors such as neuromuscular decline and reduced physical activity (PA) also play a role in accelerating the onset of sarcopenia [11]. Maximum strength capacity is reached in the third decade of life, but by the time humans reach their 50s, a steady decline has already begun [12]. Reviews of skeletal muscle aging show that the trajectory and magnitude of muscle aging differ by sex, implying women may experience some aspects of functional decline earlier [13]. Indeed, it is suggested that sarcopenia has its roots in middle age [14,15]. After age 50 years, adults lose between 5% and 10% of muscle mass per decade; given muscle mass accounts for up to 60% of body mass, this loss can exert profound effects on functional independence [16]. Sarcopenia is heavily associated with several comorbidities such as diabetes, heart disease, and obesity, as well as leading to difficulties in performing activities of daily living [17].

Existing evidence points to exercise [18-22], specifically muscle-strengthening exercise, [23-25]. Kanda et al [26] demonstrated a 12-week bodyweight resistance training program undertaken twice weekly. Participants completed 2 sets of 6 exercises (targeting thighs, lower legs, buttocks, abdomen, chest, and back) for 8 repetitions (the extremities were elevated for 3 seconds and then lowered for 3 seconds), with the sets separated by 1-minute breaks. Findings demonstrated positive effects on lower limb muscle strength in a cohort of 97 participants aged 65 years or older. Muscle plasticity is well known to persist even into the ninth decade of life, as demonstrated by Wilkinson et al [27]. Despite this, guidelines for muscle-strengthening among older adults are rarely met. According to Scottish health survey data, 14% of adults aged 75 years or older complete this exercise regularly [28]. These figures are similarly reflected across the United Kingdom (17% of men and 14% of women) [29] and the United States (22% of men and 17% of women) [30].

A potential route for bridging this muscle-strengthening gap in older adults is by harnessing digital technology [31]. One particularly promising avenue is mobile health (mHealth), which incorporates mobile apps. Approximately 90% of older adults own a laptop or computer, and in the United Kingdom, approximately 70% of people aged 60 years or older own a smartphone (approximately 67% worldwide) [32]. This indicates that older adults are increasingly digitally literate [32]. mHealth can leverage features such as push notifications, daily reminders, support, and feedback [33-36]. The capability for mHealth to deliver interventions remotely is particularly beneficial for older adults, enhancing the acceptability, efficacy, and sustainability of exercise interventions within this age group [37]. The use of mHealth represents a potentially scalable, accessible delivery mechanism that can support, augment, and reinforce muscle-strengthening behaviors when appropriately designed [38]. Using mHealth as a delivery mechanism can overcome well-documented barriers to engagement in muscle-strengthening exercise among older adults, such as access to facilities, tailored guidance, and financial barriers [39]. When grounded in behavioral theory and co-designed with older adults, mHealth interventions may offer a pragmatic means of supporting self-monitoring, motivation, and adherence, which are all critical to sustained exercise engagement. Furthermore, modern cross-platform frameworks support multiple device types (eg, smartphones, tablets, personal computers, and other web-enabled platforms such as televisions). This enables access via devices that align with preferences and existing digital practices. This reduces the risk of digital exclusion, broadens reach, and helps overcome key accessibility barriers associated with device-specific mHealth apps. However, a recent scoping review concluded there was a lack of mHealth interventions focusing on muscle-strengthening for older adults, and more research is needed to refine these approaches [40]. Furthermore, there is currently no evidence-based framework to guide the design, development, and deployment of mobile technology in older adults relating to PA or exercise [41]. Without this valuable framework, mHealth interventions in older adults could encounter unintentional barriers that may impact efficacy [42]. Of more than 350,000 health-related apps on the market [43], fewer than 2000 are designed specifically for older adults [41]. Among those, few are evidence-based or involved older adults in their design and development, and given the potential of mHealth interventions, it is prudent that further research develops a key framework to ensure future interventions are deployed effectively.

### Aims and Objectives

To address these gaps, this study represents the initial phase of a broader co-design process aimed at developing an evidence-based mHealth intervention for older adults. Co-design refers to the active involvement of end users in the creation of interventions or products and is often an approach taken in mHealth development [44]. It frequently involves focus groups, interviews, and surveys [45]. The main aim of this study was to identify key features that a muscle-strengthening exercise app should contain to enhance engagement and uptake among older adults. Secondary aims were to understand limitations of existing health apps to inform the development of a more effective app for older populations that addresses these limitations and enhances engagement. This workstream was aligned with Phase 1 (“Predesign”) of the co-design framework adapted from that described by Noorbergen et al [46].

Our specific research questions were:

- What do older adults perceive as positive aspects of current mobile apps?
- What do older adults perceive as barriers to current mobile apps?
- What features and considerations should a mobile app centered on exercise for older adults include?

In line with previous co-design approaches [47,48], the study conducted 4 co-design focus groups with older adults in groups of 6 to 8 participants, each lasting 40 to 80 minutes. The sessions were informed by a guided topic list that asked questions on general design preferences for an app interface as well as specific features of previous mHealth interventions and how these can be leveraged appropriately for older adults.

## Methods

### Overview

The study was preregistered on the Open Science Framework. Throughout the study, the COREQ (Consolidated Criteria for Reporting Qualitative Research) guidelines were adhered to [49].

### Co-Design Workflow

The study used a co-design methodology. Co-design refers to “the creativity of designers and people not trained in design, working collaboratively in the design and development process”[44]. It is a process in which researchers and stakeholders work together, focusing on incorporating end-user input into the design to ensure usability, relevance, and accessibility [44,50]. Older adult participants were involved in identifying key problems in current mHealth approaches, prioritizing app features, and refining concepts through structured group discussions and visual design prompts. Participants were encouraged to critique existing app features, propose alternatives, and discuss trade-offs of competing design options, with the aim of designing a suitable app for delivering muscle-strengthening exercise. Problem framing was achieved through older adults identifying barriers to mHealth engagement, which informed feature prioritization through consensus-building discussions. Visual design prompts were used during relevant discussions to allow participants to visualize specific features, garner discussion, and collectively refine concepts in real time. The workflow comprised 5 main stages.

### Stage 1: Preparatory Work and Recruitment

Initial preparatory work involved identifying the evidence-based mHealth features and behavior change techniques from previous app approaches and existing literature. Older adult participants with varying levels of experience with mHealth and general apps were recruited to offer diverse perspectives.

### Stage 2: Problem Framing

In the early portion of the co-design focus group discussions, participants actively shaped the problem space by discussing physical, motivational, technological, and contextual challenges relevant to aging that prevent engagement with both apps and muscle-strengthening exercise.

### Stage 3: Feature Prioritization

Participants critiqued current approaches, proposed alternative solutions, and discussed the trade-offs between usability, personalization, and engagement.

### Stage 4: Concept Refinement

Participants helped to refine proposed app concepts by suggesting changes to interface layout, notifications, app messaging content, notification timing, feedback mechanisms, and exercise delivery.

### Stage 5: Information Gathering and Translation

Following the data collection stages indicated above, findings were deductively thematically analyzed and used to shape a concrete app prototype. Insights were mapped directly to design requirements, ensuring the prototype reflected the priorities highlighted by participants. Furthermore, recommendations were established to guide future mHealth exercise intervention development for older adults.

### Study Setting and Procedures

Demographic data including, name, age, sex, occupation, education, smartphone use per day, and self-reported digital skill level were collected from participants (Table 1). A total of 2 email reminders were sent to participants prior to the focus group sessions. Four focus group sessions were completed: 2 sessions with older adults who had experience using mHealth apps and 2 focus groups with older adults without experience using mHealth apps. Co-design focus groups were chosen as the desired data collection method because they help leverage group dynamics and offer insights into shared perspectives, encouraging open discussion, which can lead to a rich qualitative dataset [51]. Each focus group session lasted approximately 40 to 80 minutes and began with an introduction to the study's purpose and the ground rules of the session. Time was then given to allow each participant to unmute themselves and give a short introduction to the group. Four focus groups were deemed sufficient because previous systematic reviews have shown that 4 sessions typically reach saturation when addressing well-defined topics in a structured format [52].

**Table 1:** Participant information.

Participant	Sex	Age (years)	Occupation	Education (ISCED^a^ [53])	Smartphone use per day	Self-reported digital skill level
1	Male	60	Retired	ISCED 6	30 minutes	Competent
2	Female	60	Retired	ISCED 6	30 minutes	Competent
3	Male	62	Local government officer	ISCED 7	3 hours	Good
4	Male	60	Supply chain manager	ISCED 6	30 minutes	Competent
5	Male	63	Retired	ISCED 8	2 hours 30 minutes	Good
6	Female	61	Retired	ISCED 3	2 hours	Competent
7	Female	63	Retired	ISCED 6	4 hours	Competent
8	Female	63	Retired	ISCED 6	4 hours	Competent
9	Female	64	Retired	ISCED 3	2 hours	Competent
10	Male	60	NHS^b^ manager	ISCED 6	1 hour 30 minutes	Competent
11	Male	74	Taxi driver	ISCED 3	2 hours	Competent
12	Male	62	IT consultant	ISCED 7	1 hour	Very good
13	Female	83	Part time student	ISCED 7	0 hours	Poor
14	Female	82	Retired	ISCED 3	3 hours	Competent
15	Female	64	Yoga teacher	ISCED 63	3 hours	Competent
16	Male	64	Fitness instructor	ISCED 6	3 hours	Competent
17	Female	61	Retired	ISCED 6	3 hours	Good
18	Female	60	Nurse	ISCED 6	5 hours	Competent

^a^ISCED: International Standard Classification of Education.

^b^NHS: National Health Service.

There were 2 semistructured focus group protocols, 1 for the experienced groups and 1 for the inexperienced groups (Multimedia Appendices 1 and 2). The protocol itself included 7 different topics (6 for experienced participants and 7 for inexperienced participants), and each topic comprised 2 to 4 questions. The inexperienced protocol included an additional topic on privacy and data sharing to delve deeper into participants’ apprehensions regarding using mHealth technology. The topics were developed based on previous mHealth intervention features [35] and consultation of the Behavior Change Technique Taxonomy version 1 (BCTTv1) [54] to gain an understanding of perceptions of common mobile app features and behavior change techniques to take forward into the development of a muscle-strengthening app. The sessions facilitated discussions using PowerPoint (Microsoft Corp) slides with images relating to app features and design, allowing participants to visualize how the app interface may look and how specific features may function within it. This resource is provided in Multimedia Appendix 3.

### Research Team and Reflexivity

All focus group discussions were designed and carried out by the lead investigator of the study (ECJB). Prior consultation with the wider research team took place to ensure all members were satisfied with the focus group structure and content. Participants in each session included some individuals who were previously known to the investigator from previous research projects, and all were made aware of the broader purpose of the study. The investigator was a novice qualitative researcher with greater expertise in quantitative methods.

### Participants and Recruitment

Participants were recruited using a combination of methods. Emails were sent to control group participants of previous studies conducted by the research team who had consented to be contacted for future studies. The Men’s Sheds organization, the University of the Third Age (U3A), and the LEAP project were contacted to ascertain interest. These organizations fundamentally exist for older adults and work in proximity to the target population and, as such, were seen as appropriate contacts. Further recruitment was conducted through social media advertising.

To be included, participants had to meet the following criteria (1) be English speaking, (2) community dwelling (not residing in a skilled nursing facility), (3) aged 60 years or older, (4) have access to a mobile device capable of downloading apps, and (5) have access to a device capable of accessing the Microsoft Teams software and understand how to use the platform. Participants were asked prior to recruitment whether they were experienced or inexperienced with mHealth or exercise apps.

We aimed to recruit approximately 20 older adults, as this number allowed us to have 6 to 10 participants in each focus group, which facilitates good discussion in which all participants can contribute. The final number of participants included was 18; a total of 2 participants withdrew because the study timeline did not align with their personal schedules. Of the 18 participants included, 9 had experience with mHealth or exercise apps and 9 had no experience. Inexperienced participants were included to ensure a highly inclusive and informed design process, with the view to increasing scalability.

### Data Collection

Each focus group session lasted approximately 40 to 80 minutes. Initially, the ground rules of the session were outlined. Each participant was asked to provide verbal consent for the session to be recorded. Each session was recorded, and raw transcripts were produced. The lead investigator checked transcripts for accuracy, grammar, and spelling. A clean verbatim approach to transcription was selected to balance accuracy with clarity by capturing the essential meaning of participants’ words while omitting unnecessary filler words, false starts, and stutters. This approach has been previously justified within focus group literature as an ideal method to ensure richness of the discussion is captured while removing excerpts that may make the transcript difficult to read [55]. Finalized transcripts were distributed to participants for member checking. All participants agreed that the transcript was an accurate depiction.

### Data Analysis

All data collection and analysis were conducted by the lead investigator (ECJB) with guidance from the wider research team. Once transcribed and validated, deductive thematic analysis was undertaken to generate themes and gain an understanding of the data [56]. As part of the analysis the Technology Acceptance Model (TAM) [57] was used as the theoretical framework to guide coding and development of themes. The TAM is a widely used framework that explains how users adopt and use technology. It suggests that 2 key factors influence technology acceptance: perceived usefulness, defined as the extent to which a person believes a piece of technology will benefit them; and perceived ease of use, defined as the degree to which a person believes that using the system will be free from effort [57].

Thematic analysis followed Braun and Clarke’s [56,58] 6-phase framework. To operationalize the TAM within the thematic analysis approach, participants' language was first coded inductively at the semantic level to ensure the codes remained grounded in their experiences and meaning. A deductive, theory-driven coding approach was then used, guided by the TAM and the study’s research questions, by mapping the codes onto the TAM constructs. Semantic coding focused on surface-level meanings to remain close to participants’ language and experiences. Codes reflecting perceived benefits, value, or relevance to participants’ health were grouped under the first main construct, perceived usefulness, while codes relating to effort, complexity, navigation, and usability were grouped under the second main construct, perceived ease of use. This allowed for the development of key themes grounded in the TAM. This 2-step process ensured participants’ language informed the analysis prior to interpretation and meant the TAM functioned as an organizing framework. This approach was appropriate given the aim to identify app features, barriers, and facilitators relevant to older adults. The analysis revealed positive aspects of current apps, limitations affecting mHealth uptake, and key design considerations to enhance usability. These insights directly informed the app refinement phase, ensuring alignment with older adults’ preferences and needs. Finally, the report was written to present a coherent narrative linking the themes to TAM and the study aims.

### Ethical Considerations

Upon expression of interest, participant information documents were shared. Participants were given the opportunity to ask any additional questions for clarity before agreeing to participate. Electronic consent forms were completed prior to the focus groups. Participants were then given details of the focus group (date and time) and emailed a meeting link along with telephone instructions on how to access the Microsoft Teams session. During the session, participants were allowed technical support from a family member or friend.

The focus groups were completed on Microsoft Teams for participant convenience; this also allowed a broader geographical sample. Participants joined the Teams session using the meeting link that had been emailed to them previously and using their own smartphone, tablet, laptop, or computer device. Each participant was asked to provide verbal consent for the session to be recorded for subsequent thematic analysis. Participants were not provided with any financial or other form of compensation for their participation. All data were treated confidentially and anonymized using participant identification numbers. Focus groups were video recorded online (with all participants’ permission) to facilitate subsequent thematic analysis. Recordings and associated data were stored on password-protected computers available only to the principal investigator (ECJB).

This study was carried out in accordance with the Declaration of Helsinki and approved by the University of the West of Scotland Health and Life Sciences Academic Integrity and Ethics Committee (approval number 22512).

## Results

### Overview

A total of 4 main themes were identified from the analysis, with several subthemes (Table 2). These themes encompass the previous lived experience of participants with mHealth technology, areas in which participants have struggled with engagement, and key considerations to take forward to enhance usability and intervention effectiveness. Full excerpts from focus group discussions are included in Multimedia Appendix 4.

**Table 2:** Four main themes and the corresponding subthemes derived from thematic analysis.

Theme	Subtheme
Theme 1: mHealth^a^ as a tool for supporting health and well-being	Overcoming barriers to accessibility Potential for educational support via mHealth
Theme 2: motivation and engagement through app features	Progress tracking as a source of motivation Rewards and streaks as reinforcement
Theme 3: drawbacks of current mobile apps	Complexity and poor usability Skepticism over effectiveness, safety, and evidence base
Theme 4: desired app elements and features	Customizable reminders Clear video demonstrations of muscle-strengthening exercise Customizable exercise options and feedback Preferences regarding social features of mHealth

^a^mHealth: mobile health.

### Theme 1: mHealth as a Tool for Supporting Health and Well-Being

This theme encapsulates participants perceptions of mHealth to support health and well-being in their daily lives. Across both experienced and inexperienced users, mHealth technologies were understood to be mechanisms that help promote health behavior through increasing awareness, providing feedback, and prompting action.

Participants who were experienced with mobile technology drew upon their experiences of mHealth promoting activity via push notifications and how advanced features support them during exercise. Experienced participants highlighted that feedback mechanisms and prompts help to break long bouts of sedentary behavior and regulate activity, as one participant noted:

A part of my app. It tells you if you’ve been sat for a long time and it just basically says maybe it’s time to get up and move.

Participant 6

These features were perceived as highly important in the context of aging, where participants noticed a steady decline in their own activity and had a desire to maintain functional independence.

Instant feedback features such as heart rate and activity summaries were described as reassuring, motivating, and a useful mechanism for tracking exercise effort and intensity with greater confidence. The theme highlights a broader understanding, primarily among experienced participants, of their own deterioration during the aging process and that mHealth interventions may encourage them to participate in exercise, which would attenuate their decline.

This first theme led to the development of the subtheme, overcoming barriers to accessibility. The subtheme broadens understanding of the challenges older adult participants have faced in relation to muscle-strengthening exercise, namely physical and financial limitations. Participants identified digital resources as a means of overcoming these barriers. One participant noted:

I used to go to YouTube. I’ve discovered fabulous 50s. For people who are over 50 and then it’s just as a range of different exercises, you know, usually they’re just walking included in it, and it means you can do it in the house or whatever.

Participant 8

Furthermore, an experienced participant reflected that an app aid accessibility by stating:

Rather than expecting people to have, you know, weight sets or whatever get, you know, used items that people have readily accessible round their houses.

Participant 1

A further subtheme identified in relation to Theme 1 was the potential for educational support via mHealth. Participants consistently emphasized the potential for mHealth technologies to provide educational support related to exercise benefits, technique, and progression. Experienced participants drew upon the self-quantifying nature of digital exercise tools, acknowledging the empowerment of personal metrics, which encouraged continued engagement. Participants highlighted the value of clear guidance to support learning at their own pace:

I’ve not got the sort of strength that I probably want to have and just want to sort of educate myself into what the next steps could be just to sort of bring myself on.

Participant 4

This subtheme highlights the understanding that this technology has the potential to educate older adults on muscle-strengthening exercise.

### Theme 2: Motivation and Engagement Through App Features

This theme captures how mHealth features were perceived to influence motivation, routine development, and sustained engagement with exercise behavior. Participants described how features such as prompts, feedback, and reinforcement mechanisms supported adherence by fostering accountability and a sense of progress.

Participants drew upon familiar examples from fitness apps that use nudge reminders to increase engagement. Similarly, experienced participants highlighted the benefit of wearable and app technologies that use haptic nudges to prompt users to break up sedentary time and send motivational messages to urge users to meet step goals. These features were perceived as supportive, with one participant stating:

That’s what I like about it. I just realised I got a reminder today that I haven’t done my third run of the week, so that’s good.

Participant 3

Personalized feedback on goal progression was also viewed as central to maintaining engagement.

A subtheme generated from Theme 2 was progress tracking as a source of motivation. This subtheme identified motivators and features that engage participants, such as the ability to track their progress. Participants highlighted that tracking their own progress when working toward an activity goal and recording improvements motivated and encouraged them to continue. Inexperienced participants highlighted that progress tracking could provide reassurance and validation.

A final subtheme developed as part of Theme 2 was rewards and streaks as reinforcement. This subtheme encapsulates experienced participants’ thoughts, feelings, and encounters with reward systems (ie, gamification systems). Participants expressed mixed views, highlighting a clear trade-off between motivation and engagement. For some experienced participants, rewards such as badges, medals, and streaks were described as powerful motivators for sustained engagement:

I got fanatical about continuing my streak.

Participant 12

In contrast, those who had little experience with these features viewed the prospect of gamification as childish and condescending. These participants felt that simplistic rewards such as badges undermined autonomy and preferred personalized meaningful feedback linked to functional improvement. This highlights the challenge of designing engagement features that accommodate varying levels of digital familiarity and differing motivational preferences.

### Theme 3: Drawbacks of Current Mobile Apps

The third theme reinforced the key issues that older adults face when engaging with mobile apps. Across both experienced and inexperienced users, drawbacks were not described as isolated issues but as indicators that mobile apps in their current form are inadequately designed for older adults’ needs and priorities. Participants highlighted a general reluctance to input personal information, reflecting broader concerns around privacy and emotional burden of mHealth. Some participants described negative experiences with continuous monitoring devices, such as sleep tracking, which were perceived to exacerbate anxiety rather support well-being:

I’ve got a problem with sleep trackers from work perspective. They induce more anxiety than they help to be perfectly honest.

Participant 10

These sentiments highlight broader skepticism among participants about whether app engagement would meaningfully enhance health. Mainly inexperienced participants also felt that existing apps lacked relevance for older adults. Current apps were described as cluttered and filled with unnecessary features, creating confusion and discouraging sustained use. Several participants noted that while health and fitness apps appear abundant, those targeting muscle strength while also catering to age-related needs are scarce, further highlighting the accessibility gap between older adults and app design.

A subtheme that subsequently developed was complexity and poor usability. This subtheme explored specific difficulties participants encountered when engaging with mobile apps. Experienced participants described current fitness apps as being overcomplicated and overloaded with information, which they found hard to understand. Inexperienced participants felt that even when they have attempted to use currently available apps, they found them time-consuming and burdensome. Older adults indicated that simplicity was key and expressed a preference to engage only with activities and information that were personally relevant. Interestingly, even those who self-identified as digitally competent felt that current exercise apps were complex and difficult to use effectively without becoming overloaded with information.

A further subtheme that developed was skepticism regarding effectiveness, safety, and evidence base. Participants expressed widespread skepticism regarding the effectiveness and safety of current exercise apps, particularly for older adults with musculoskeletal and chronic conditions, such as sarcopenia. Despite recognizing the importance of strength training, sometimes informed by professional health care practice (eg, nursing), some participants questioned whether current apps were evidence-based or capable of safely supporting strength improvements. Concerns were also raised about the credibility of online exercise content. As one participant noted:

Now I look at a lot of videos and I think these people are promoting that and it’s dangerous things they are doing, you know, when they’re promoting it for older folk.

Participant 17

These concerns contributed to hesitation around adoption and highlighted the importance of clinical credibility and age-appropriate design in future mHealth interventions.

### Theme 4: Desired App Elements and Features

The final theme identified was desired app elements and features. This theme was developed in conjunction with the positive and negative aspects of current mobile apps. It synthesizes participants’ perspectives on how mHealth interventions should be designed to support sustained engagement and safety among older adults. Rather than describing isolated feature requests, participants articulated broader design priorities centered on simplicity, autonomy, and relevance to older adults’ needs.

Participants consistently emphasized the importance of simple interface design. Features such as clear navigation, high-contrast colors, and adjustable font sizes were described as essential for reducing cognitive load. Participants described positive experiences with apps and websites that prioritized clarity over complexity, reinforcing the need to accommodate age-related sensory decline. One participant noted:

Some of them are better than others, you know, and most of them are kind of intuitively simplistic. So probably like those, not too busy and easy to follow. Is the kind of rule of thumb.

Participant 10

A subtheme that developed in conjunction with Theme 4 was customizable reminders. Personalization emerged as a central mechanism for supporting sustained engagement. Participants described a strong preference for control over reminder frequency and timing, highlighting the importance of aligning prompts with individual routines. The timing of notifications was encapsulated within this subtheme, with many experienced and inexperienced participants stating that a reminder in the morning to engage in exercise would suit their routine. One participant noted that it was important for this reminder to come at the start of a new week. However, counterpoints were raised, with 1 participant stating that they often find their life is busy in the morning and, as such, a reminder later in the afternoon would be beneficial because they would have more time to engage in exercise at this time of day. This subtheme highlights autonomy as a key design principle, with flexibility enabling mHealth interventions to accommodate diverse lifestyles.

A further subtheme generated was clear video demonstrations of muscle-strengthening exercise. When discussing the specific features related to enhancing usability, both experienced and inexperienced participants emphasized a need for clear video demonstrations to improve safety and effectiveness. Some participants highlighted the importance of being able to take the learning process at their own pace and felt it would be useful to have the option of pausing, fast-forwarding, and rewinding a video demonstration to ensure they could adequately perform the exercise. Furthermore, participants who had experience using similar apps for gym-based exercise recalled that being able to click on certain exercises with which they were unfamiliar and access video demonstrations helped them build confidence with the exercise. Additionally, all participants emphasized the ability to resonate with the video demonstrations, suggesting the demonstrator in current available apps is often much younger. The consensus was that if the demonstrator within the videos were an older adult, this would add a sense of realism with which users could relate.

A further subtheme developed in relation to Theme 4 was customizable exercise options and feedback. Autonomy over exercise selection and progression was viewed as essential for maintaining engagement. Participants built on this idea by highlighting that users have a range of preferences for muscle strengthening–based exercise, and allowing them to select the activity may enhance their experience. Discussion developed further to generate the idea of customization to allow users of different abilities to be signposted to exercises appropriate for them. Participants described frustration with one-size-fits-all approaches commonly experienced by older adults in gym and digital environments. A staged structure, such as tiered programs guided by front-loaded questioning, was identified to personalize exercise without requiring users to label or compare themselves to others. Participants noted that inappropriate labeling may negatively impact confidence if their own progression stalled. However, participants appreciated that their app experience would be personal to them and that their individual needs would be catered to.

A final subtheme developed in relation to Theme 4 was preferences regarding social features of mHealth. Discussion surrounded the notion of including social features within the app to allow participants to complete exercise together with family or friends. A few experienced participants underlined that they felt the option to exercise together with peers would enhance their motivation, as they often find it hard to exercise alone. Others felt social features were unnecessary, as they become competitive and distract from the initial goal of encouraging exercise. The general feeling was that having the option to engage in social aspects would be beneficial but should be secondary to encouraging the uptake of strengthening exercise. Insights gathered particularly in this theme directly informed app prototype development, ensuring older adults’ identified needs were translated into design features.

## Discussion

### Overview

The aim of the focus groups was to determine essential features that a muscle-strengthening exercise app should include to improve engagement among older adults. This study examined the limitations of current mHealth apps that hinder effectiveness and contribute to low adoption. During deductive thematic analysis, the research questions were considered alongside the TAM framework, which helped to produce 4 interrelated themes and 10 subthemes. Participants’ insights highlighted their nuanced experience of both mHealth technology and muscle-strengthening exercise. The development of these main themes helps to build on broader practical implications and recommendations for the future of muscle-strengthening app development for older adults, contributing to healthy aging.

This manuscript represents a significant contribution to the field of digital health and aging through its originality, significance, and methodological rigor. It introduces a transformative approach by applying a co-design methodology to the development of a muscle-strengthening mHealth intervention for older adults, a population historically underserved in app design. We believe this is the first study to commence co-design of a muscle-strengthening mHealth intervention for older adults. The integration of the TAM into thematic analysis offers a novel theoretical lens for interpreting older adults’ engagement with digital exercise tools, advancing conceptual understanding from gerontology to digital health and beyond.

This study’s significance lies in its potential to reshape how mHealth interventions are designed and deployed for older populations. By directly involving older adults with varying levels of digital literacy and app experience, the research generates actionable insights that challenge assumptions about usability, motivation, and accessibility. The findings have immediate practical implications for developers, clinicians, and policymakers seeking to address sarcopenia and promote healthy aging through scalable, evidence-based digital solutions. Methodologically, the study demonstrates exceptional rigor. The sampling strategy ensures representation across digital experience levels, and saturation is robustly evidenced. The alignment of findings with TAM constructs provides a coherent analytical framework, and the study is transparently reported in accordance with the COREQ guidelines.

### Theme 1: mHealth as a Tool for Supporting Health and Well-Being

The first theme conveyed participants’ views on how mHealth technology can support daily health and well-being. Although direct experience varied across focus groups, participants generally appreciated its potential to encourage healthier behaviors and improve access to PA, particularly muscle-strengthening exercise.

Those with experience described how mHealth guided healthier behavior and increased awareness of health metrics. These findings align with nudge theory, a concept in behavioral economics suggesting that positive reinforcement and indirect suggestions can influence behavior [59]. Nudge theory has been applied in studies using mHealth to promote PA in older adults [60-62]. For example, Yamada et al [61] showed that tablet-based nudges encouraged healthier behaviors in older men. Other research has similarly demonstrated that mHealth features can be leveraged to support health and well-being in older adults. Kwan et al [63] trialed a brisk walking intervention with 33 participants using the Samsung Health app and WhatsApp, delivering programs, reminders, and rewards. The intervention enhanced moderate-to-vigorous PA uptake by 86 minutes per week post intervention, supporting consensus in the literature that mHealth promotes health behavior.

Older adults often face accessibility barriers to exercise [64], a theme echoed by participants. Research has identified extrinsic barriers such as lack of transport, supervision, or facilities, and intrinsic barriers including perceptions of being too old, fatigue, or fear of injury [65]. Numerous studies have shown that digital delivery can help reduce such barriers [66-68]. Sciammana et al [69] delivered a 24-week, 1-minute bodyweight intervention via weekly emails to 24 older adults with a mean age of 71.6 (SD 8.9) years. The findings outlined that 75% of participants completing at least half of the prescribed sessions. Extending such approaches to mHealth, with an evidence-based approach, may allow for enhanced intervention delivery methods and further increase accessibility and adherence in muscle-strengthening contexts.

A further consideration from participants was the suggestion of incorporating household items, such as filled bottles or shopping bags, into mHealth exercise interventions to overcome limited gym access. While this could provide a training stimulus [70], safety concerns arise, as equipment use increases injury risk, especially given that most older adults lack resistance training experience [71,72]. Thus, equipment-free exercises may be more appropriate for mHealth resistance programs in older adult contexts.

Participants also valued the educational potential of mHealth. They emphasized the need for simple apps providing essential information to facilitate muscle-strengthening without unnecessary features. This reflects evidence on the importance of informational support in behavior change interventions for older adults [73,74]. Concerned about age-related muscle loss, participants welcomed resistance training and mHealth initiatives but highlighted a knowledge gap that left them ill-equipped to train independently. Prior research has also identified poor understanding of resistance training’s purpose and safe practice among older adults [66]. Despite guidelines recommending twice-weekly strength training [75], messaging is often vague [76]. mHealth could bridge this gap by offering educational support, such as video demonstrations, to enable safe and effective training [40].

Overall, this theme underscores mHealth as a promising avenue for promoting muscle-strengthening behavior change. Participant insights link closely to the construct of perceived usefulness, which remains a strong predictor of mHealth engagement, particularly when technology supports daily life and independence [77].

### Theme 2: Motivation and Engagement Through App Features

A crucial determinant of engagement with mHealth among older adults was the presence of motivational app features. Participants consistently discussed elements such as prompts, reminders, and progress feedback, explaining how these supported the integration of health behaviors into daily routines. These features were perceived to provide structure for engaging in PA and other health-related behaviors. Specifically, prompts and reminders acted as “nudges” that encouraged consistent PA engagement and supported long-term behavior change, aligning with broader evidence identifying these functions as key to sustained mHealth use [78,79].

Participants highlighted reminder functions, typically delivered via push notifications, as behavioral cues promoting consistent app use and engagement in health behaviors. These findings correspond with literature demonstrating the effectiveness of prompts for PA promotion, particularly among older adults who benefit from external structure [80,81]. Reminders and reinforcement messages can enhance exercise adherence when they become integrated into users’ routines [82,83].

Progress tracking emerged as another major motivator. Both experienced and inexperienced users described viewing their progress over time as encouraging and essential for maintaining motivation. This reflects prior work emphasizing the value of feedback and progress monitoring in behavioral interventions [84]. For instance, Geraedts et al [85] combined wearable sensors and tablet-based strength exercises, enabling participants to view improvements, which enhanced motivation. Such findings relate to the concept of self-quantification, which refers to the practice of methodically collecting, measuring, and analyzing personal data using technology [86], wherein monitoring one’s data fosters engagement and motivation [87].

While mHealth interventions targeting aerobic activity benefit from automated data collection via wearables [63,88,89], muscle-strengthening interventions face greater challenges. Accurately capturing resistance or repetitions often requires manual input, increasing user burden. Nonetheless, the motivational benefits of visualizing progress may justify this effort if it enhances adherence [90].

Views on rewards and streaks were mixed. Participants familiar with such features viewed them positively, while inexperienced users often perceived them as “condescending.” Although these negative attitudes may stem from unfamiliarity [91], the literature supports the effectiveness of gamified features, particularly in step-based interventions [92-94]. It may therefore be beneficial to personalize gamification for older adults. Although studies have shown the general benefits of gamification [95-97], these features must be context-sensitive and tailored to older users. Moreover, step-based gamification relies on automated tracking, whereas applying similar approaches to strength training poses technical and usability challenges. Streak-based systems may also risk shifting focus away from meaningful engagement by becoming the main focus of the user rather than the exercise intervention at hand [98].

These findings connect closely with the TAM construct of perceived usefulness, a key predictor of intention to use. The theme encapsulates features that translate app functions into tangible benefits by motivating users to exercise or track health behaviors. When older adults perceive clear value in how an app supports their activity or progress, they are more likely to adopt and sustain its use [99]. Overall, this study highlights the importance of motivational features while urging careful, context-aware integration of gamification in mHealth design for older adults.

### Theme 3: Drawbacks of Current Mobile Apps

The third theme highlighted several disadvantages older adults face that hinder uptake and sustained engagement with mHealth technology. Data from this study identified drawbacks such as poor usability, irrelevant content, and doubts regarding safety and effectiveness—issues echoed in the broader literature as key barriers to mHealth adoption among older adults [100]. Participants, particularly those inexperienced with apps, felt current designs often neglect older adults’ needs. A perceived lack of personal relevance reduced engagement, consistent with research on the poor alignment between mobile app design and older users [101]. Participants also noted limited accessible options for home-based muscle-strengthening, with most apps failing to account for reduced physical capacity [102]. Moreover, distrust in tracking technologies emerged as a barrier, consistent with existing findings on digital health skepticism in this demographic [103].

Complex app interfaces were another major source of frustration. Even digitally confident participants described current exercise apps as “too advanced” or “overloaded with information.” This aligns with evidence identifying cluttered design and poor navigation as key deterrents for older users [101,104]. While design guidance often focuses on elements such as button size, textual and navigational clarity remain neglected [101]. The experiences in this study reinforce the need for simplified navigation, clear signposting, and concise exercise programming to reduce user burden and flatten the learning curve—particularly for those wary of technology [105].

Participants also expressed concerns about safety and effectiveness, particularly for unsupervised, home-based exercise. Many questioned whether current or future apps could ensure correct technique and injury prevention—concerns amplified among those with chronic conditions such as osteoporosis or osteoarthritis. Experienced users further noted the risks of non–evidence-based exercise advice, contrasting such apps with the reassurance of supervised exercise. These concerns, rooted in participants’ experiences, align with literature highlighting trust as a critical factor in mHealth adoption [106]. The lack of a transparent evidence base underpinning many apps remains a key limitation [107] and may particularly discourage those with long-term conditions who fear injury [108].

Participants also questioned the credibility of some exercise apps, noting that a lack of visible evidence base or endorsement from recognized organizations undermines trust [42]. The perceived absence of personalization and adaptive programming reinforced the need for tailored content and clear signposting of safe options for older adults.

Negative prior experiences and limited understanding of strength training principles led some participants to doubt apps’ ability to accurately track progress. Unlike aerobic activity, which can be easily quantified via automated measures such as step counts or heart rate, tracking strength improvements often requires manual input, increasing user burden [105]. Simplifying self-reporting—for example, logging completed sets rather than repetitions or using automated sensing technologies such as accelerometers—could reduce this burden [109,110]. However, the cost of smart resistance devices poses a scalability issue [111], limiting feasibility given that mHealth interventions are intended to reduce, not increase, costs [112].

Overall, these findings correspond to the TAM constructs of perceived usefulness and perceived ease of use. Apprehension regarding safety, evidence base, and design complexity reduced participants’ belief that exercise apps can effectively support health and well-being. Such perceptions lower both perceived usefulness and ease of use—key predictors of technology adoption [113]. Consequently, addressing these design and trust-related drawbacks will be essential to enhance older adults’ acceptance and engagement with future mHealth interventions.

### Theme 4: Desired App Elements and Features

The final theme highlights actionable recommendations for future mHealth intervention development in older adults, broadening the understanding of muscle-strengthening apps for this population. These findings help direct researchers and app developers to enhance uptake and long-term engagement with muscle-strengthening exercise and broader mHealth interventions [114].

The focus group discussions reinforced that app usability is a central priority for older adults. Participants emphasized that many existing mHealth resources do not adequately address their needs, which negatively influences uptake and sustained engagement. Clear navigation, a simplified and uncluttered interface, and high-contrast color schemes were identified as key design features that would enhance usability. Participants drew on previous negative experiences with apps featuring unnecessary functions, visually dense layouts, and small text, aligning with literature underscoring the importance of inclusive digital design [115]. Font size was also highlighted as a critical consideration; although text can be adjusted within device settings, ensuring an appropriate default size may improve perceived ease of use, as simpler interfaces can reduce cognitive load and support engagement among older adults [116].

Participants emphasized that personalization should be a core component of the app experience. Customizable reminders were viewed as essential, with participants noting that reminder frequency and timing should align with individual routines to prevent disengagement. This reflects previous findings that excessive notifications can contribute to technology-related stress and reduced adherence among older adults [117]. As also highlighted in Theme 2, the timing and tone of prompts were considered crucial: participants generally preferred a single reminder on the morning of an exercise day, delivered in supportive and nonjudgmental language. This aligns with literature demonstrating the importance of encouraging tones in digital prompts for older adults [118]. Evidence from Wolner-Strohmeyer et al [119], who conducted a 12-week PA intervention with adults aged 60 years or older, showed that twice-weekly reminders significantly increased activity levels, further supporting the value of well-calibrated reminders. Consistent with these findings, participants suggested that one appropriately timed reminder would be sufficient. Consequently, future app development should prioritize adaptable reminder systems that can be tailored to users’ daily routines to enhance engagement.

Participants expressed a preference for greater autonomy within the muscle-strengthening program, including the ability to select exercises and create personalized routines. They believed that having control over their workouts would increase enjoyment, support longer-term engagement, and allow better alignment with individual physical abilities. This aligns with evidence from previous mHealth research demonstrating that autonomy can enhance adherence. For example, Mair et al [35] reported improved adherence (67%) when participants set their own step and activity goals, while Daniels et al [120] observed a 135% adherence rate in a 5-week intervention where users selected activities and customized workouts—although this figure reflects adherence relative to self-chosen goals. However, such customization is more readily implemented in aerobic-based interventions; strength-based programs present additional challenges regarding safety and effectiveness [121]. Limited knowledge of resistance training in older adults [122] further increases the risk of unbalanced or unsafe exercise choices. Additionally, providing too many options may increase cognitive load, adding to the usability burden identified in mobile app design guidelines [101].

A key message from these discussions was the need for varied entry points in strength programs based on individual capacity. Older adults are not a homogenous group [102], and age-related conditions amplify disparities in musculoskeletal health. Sarcopenia prevalence rises from 5%-13% in adults aged 60-70 years to around 50% in those aged 80 years and older [123], with substantial declines in muscular capacity; for example, leg press 1-repetition maximum dropping from 237 kg in adults aged 60-64 years to 172 kg in those aged 80-85 years in men [124]. These findings highlight the need for structured difficulty levels within mHealth apps, enabling participants to select a starting level aligned with their ability.

Participants stressed the need for instructional clarity through clear video demonstrations of muscle-strengthening exercises. These calls were rooted in safety and confidence in the exercise materials and were an extension of the previous discussion encapsulated in Theme 3. Participants valued video demonstrations due to their accessible features (pause, rewind, and replay), emphasizing that older adults feel it is important to learn muscle-strengthening exercises at their own pace. These specific needs have been catered to in previous interventions, which have demonstrated the benefits of using video demonstrations to assist the provision of muscle-strengthening exercise for older adults [125,126]. These findings also link to the broader literature, which highlights that older adults value personalized learning and the ability to revisit material as needed [127]. A crucial consideration that emerged from discussing the provision of video demonstrations was the demand from participants that videos should be relatable, ideally carried out by an older adult with a relatable physique. Participants felt that having an older adult demonstrate the exercise would help them resonate better and reinforce that muscle-strengthening exercise is not unattainable. These findings are comparable with broader literature, which outlines how age-related promotion of exercise can help foster identification in older adults and enhance engagement [128].

Participants had mixed views on social features of apps. While some felt group-based exercise or peer support would boost their motivation to engage, other participants felt that exercise was a personal experience and did not want social exposure. These thoughts are mirrored in the literature; a previous review by Tong and Laranjo [129] highlighted that while some participants benefit from social features in mHealth activity interventions, many are concerned about direct comparisons to peers, which may become off-putting. The consensus from participants was that social features should be included but optional rather than a default requirement, thereby catering to all needs. The findings from this study link to older adult literature, which highlights that social connectedness can help boost long-term adherence to exercise programs [130]; however, imposing this in an mHealth intervention risks alienating some participants.

The fourth theme is clearly rooted in both TAM key constructs of perceived usefulness and perceived ease of use. The emphasis on simple navigation and high-contrast visuals supports perceived ease of use by reducing the physical and cognitive requirements to engage with the app. Meanwhile, personalization of reminders, exercise selection, and instructional clarity enhances participants’ perceived usefulness, ensuring that the app can deliver meaningful benefits.

### Strengths and Limitations

One key strength of the study is that the findings offer insight into the applicability of mHealth interventions for muscle-strengthening among older adults. The findings provide a perspective on older adults’ needs, wants, thoughts, and feelings regarding engagement with mobile technology, which in turn may enhance participation. By mapping the themes to the TAM core constructs of perceived usefulness and perceived ease of use, the study offers a clear understanding of the factors influencing mHealth uptake in older adults and presents a framework embedded within a broader co-design process. To the best of the authors’ knowledge, this is the first study to outline specific design and intervention recommendations exclusively for muscle-strengthening mHealth interventions for older adults. A further strength of this study was the representation of both experienced and inexperienced older adults in engaging with mHealth technology. This helps guide future interventions to ensure they cater to all needs and maximize intervention effectiveness. Moreover, the inclusion of both males and females ensured diverse perspectives and key design considerations.

The study is not without limitations, primarily recruitment bias. The sample was recruited mainly from the central Scotland area, and the majority were well-educated. Participants’ affluent backgrounds likely reduced diversity, which may limit the applicability of the framework to the broader population. Furthermore, due to participants’ affluence, it is likely they had a baseline digital literacy higher than that of older adults of lower socioeconomic status [131,132].

Participants in this study may have been more likely to complete exercise, as they may have been attracted to participate in this research. This is therefore not representative of the broader older adult population, in which fewer than 2% to 20% regularly complete muscle-strengthening exercise [29,133]. The study did not formally assess participants’ baseline activity levels or adherence to muscle-strengthening guidelines; therefore, it was not possible to contextualize participants’ perceptions or completion of current PA guidelines. The sample may have been more health-conscious or physically active than the general older adult population. This limitation should be considered when interpreting findings, as attitudes toward mHealth and exercise may differ among the more physically inactive individuals.

Moreover, the age profile of the participants largely reflects the “young-old” older adult population [134]. The mean age of the sample was 65 years, and participants were generally healthier and potentially more digitally capable than anticipated from older age groups. This may limit the transferability of these findings to older adults living with frailty, multimorbidity, or cognitive impairment.

### Practical Recommendations

In line with the principles of the co-design process [44], this study points to several key design considerations for future mHealth interventions aimed at supporting muscle-strengthening exercise in older adults. These can be distilled into five guiding recommendations (1) simplicity and clarity in interface design; (2) personalization of exercise content, reward functions, and reminders; (3) safe and relatable instructional resources, such as video demonstrations with older adult demonstrators; (4) flexible exercise customization; and (5) optional social features.

### Conclusion

Overall, this study aimed to identify key features that a muscle-strengthening exercise app should include to enhance engagement and uptake among older adults and to examine the limitations of existing health apps to inform more effective design. Findings highlight both positive and negative aspects of current apps, as well as essential considerations for future development. Across 4 themes, participants recognized the potential of mHealth to support health and well-being, identified motivational features, outlined usability challenges, and proposed desired app elements. Design preferences included large font, clear navigation, personalized exercise options, customizable reminders, instructional videos, and sensitivity to age-related needs. These insights offer a user-informed framework for app refinement. In conclusion, this study provides a foundation for impactful mHealth design targeting muscle-strengthening and sarcopenia prevention in older adults, with the potential to inform future interventions and improve digital health equity.Top of Form

## Data Availability

The datasets generated or analyzed during this study are available in the Open Science Framework repository.

## Appendix

Multimedia Appendix 1 Experienced question list.

Multimedia Appendix 2 Nonexperienced question list.

Multimedia Appendix 3 Codesign focus group PowerPoint.

Multimedia Appendix 4 Quote table.

## Abbreviations

BCTTv1: Behavior Change Technique Taxonomy version 1
COREQ: Consolidated Criteria for Reporting Qualitative Research
mHealth: mobile health
PA: physical activity
TAM: Technology Acceptance Model
U3A: University of the Third Age
