# Supplementary material for: Designing an mHealth App to Encourage Uptake of Muscle-Strengthening Exercise in Older Adults: Co-Design Focus Group Study
Source: JMIR Aging. 2026 Mar 12;9:e87332. doi: 10.2196/87332 (PMC13022557; doi:10.2196/87332)
Supplement: Multimedia Appendix 1 [file aging_v9i1e87332_app1.docx]

**Topic and Question List**

Starter Discussion: 10 minutes

1. What is your experience of digital technology on a daily basis?
2. Do you currently use mobile apps?
3. What are positive features of current mobile applications you use?
4. What are weaknesses/barriers to use of current mobile applications?
5. How have these barriers affected your use of mobile applications?
6. What is your experience of physical activity or exercise?
7. On a scale of 0-100 how important is physical activity or exercise to you?

Exercise Applications:

1. What exercise apps have you used before?
2. What do you like most about the app?
3. Your least favourite functions?
4. What key features would you like to see in a mobile application centred on exercise?
5. What specific design elements (e.g., font size, button placement) would improve the usability and accessibility of the app?
6. What types of exercise content do you find most engaging (e.g., videos, written instructions, live classes) and how important is it for the app to offer a variety of exercise options?

Self-monitoring:

1. How would you feel about monitoring your physical activity/exercise through an app?
2. How important is it for the exercise app to integrate with other tools or devices (e.g., fitness trackers, smartwatches) and what integrations would be most useful to you?
3. What metrics would you like to see?

Feedback:

1. How would you feel about receiving individualised feedback on your exercise behaviour?
2. How do you prefer to receive feedback or encouragement from the app (e.g., notifications, badges, progress tracking)?
3. What is your least favourite method of obtaining feedback/encouragement?

Goal Setting:

1. What physical activity/exercise goals would you like to set and how could an app help with this?
2. How important is it for an exercise app to offer customisation options (e.g., tailored workouts, adjustable difficulty levels)?

Reminders:

1. How frequent would you like reminders via the app?
2. How would you like to receive reminders? (push notifications etc.)
3. Which time of day would be best to receive reminders?

Social Aspects:

1. How important is it for the app to include social or community features (e.g., forums, group challenges)?
2. What type of support (e.g., virtual coaching, customer service) would you expect from an exercise app?
