# Supplementary material for: Designing an mHealth App to Encourage Uptake of Muscle-Strengthening Exercise in Older Adults: Co-Design Focus Group Study
Source: JMIR Aging. 2026 Mar 12;9:e87332. doi: 10.2196/87332 (PMC13022557; doi:10.2196/87332)
Supplement: Multimedia Appendix 2 [file aging_v9i1e87332_app2.docx]

**Topic and Question List**

Starter Discussion: 10 minutes

1. What do you currently use digital technology for (e.g smartphone, tablet, laptop, computer)? If not, why not?
2. Do you currently use mobile apps? If not, why not?
3. What is your experience of physical activity or exercise?
4. What factors encourage and discourage you from being active on a day-to-day basis?
5. On a scale of 0-100 how important is physical activity or exercise to you?
6. Do you use mobile apps to help you exercise? If so, what do you find useful and if not, why not?

Exercise Applications:

1. What types of physical activity are you currently doing?
2. How do you know what is appropriate and where do you get physical activity information?
3. What features would make a mobile app helpful for exercise? For example, would it be useful if the app could track progress, remind you to exercise or track sleep?
4. What features would put you off using a mobile app to help you exercise more?
5. What would make it easy for you to use the app? (e.g., font size, button placement).
6. How do you prefer to get instructions for exercises? Do you like watching videos, reading instructions, or having someone guide you step by step? How important would it be for the app to have different types of exercise options?

Self-monitoring:

1. How would you feel about monitoring your physical activity/exercise through an app?
2. What type of information would you like to see would you like to see – HR, repetitions, progressions for specific exercises?

Feedback:

1. Would it be helpful if you could use an app to keep track of exercise and individualise the experience for you?
2. With regards to tracking, how important is being able to see your progress over time? Would you like simple feedback on this or more complex information?
3. How would you feel about in app rewards? For example, badges or streaks for the number of days you complete the given exercise dose?

Goal Setting:

1. How could an app help you with your physical activity goals?
2. How important is it for an exercise app to offer customisation options (e.g., tailored workouts, adjustable difficulty levels)?
3. With regards to customisation, how simple or complex would you like this to be? For example, beginner, intermediate, advanced levels or a complex breakdown of reps and sets?

Reminders:

1. How frequent would you like reminders via the app? For example, once a day if you haven’t logged a workout by the time you usually would?
2. Would you like to receive reminders? (push notifications etc.) Further to this, how would you feel about the app giving you nudges of encouragement to complete more exercise throughout the day or encouragement to keep a streak of exercise going over a number of days/weeks?
3. Which time of day would be best to receive reminders? For example, would it be useful in the morning/afternoon/evening?

Social Aspects:

1. How important is it for the app to include social or community features (e.g., forums, group challenges)?
2. What type of support (e.g., virtual coaching, customer service) would you expect from an exercise app?

Privacy and Data Sharing:

1. How important is it that your exercise data is kept private?
2. Would you have any concerns relating to privacy with this kind of app?
