# Supplementary material for: Designing an mHealth App to Encourage Uptake of Muscle-Strengthening Exercise in Older Adults: Co-Design Focus Group Study
Source: JMIR Aging. 2026 Mar 12;9:e87332. doi: 10.2196/87332 (PMC13022557; doi:10.2196/87332)
Supplement: Multimedia Appendix 3 [file aging_v9i1e87332_app3.pptx]

## Slide 1
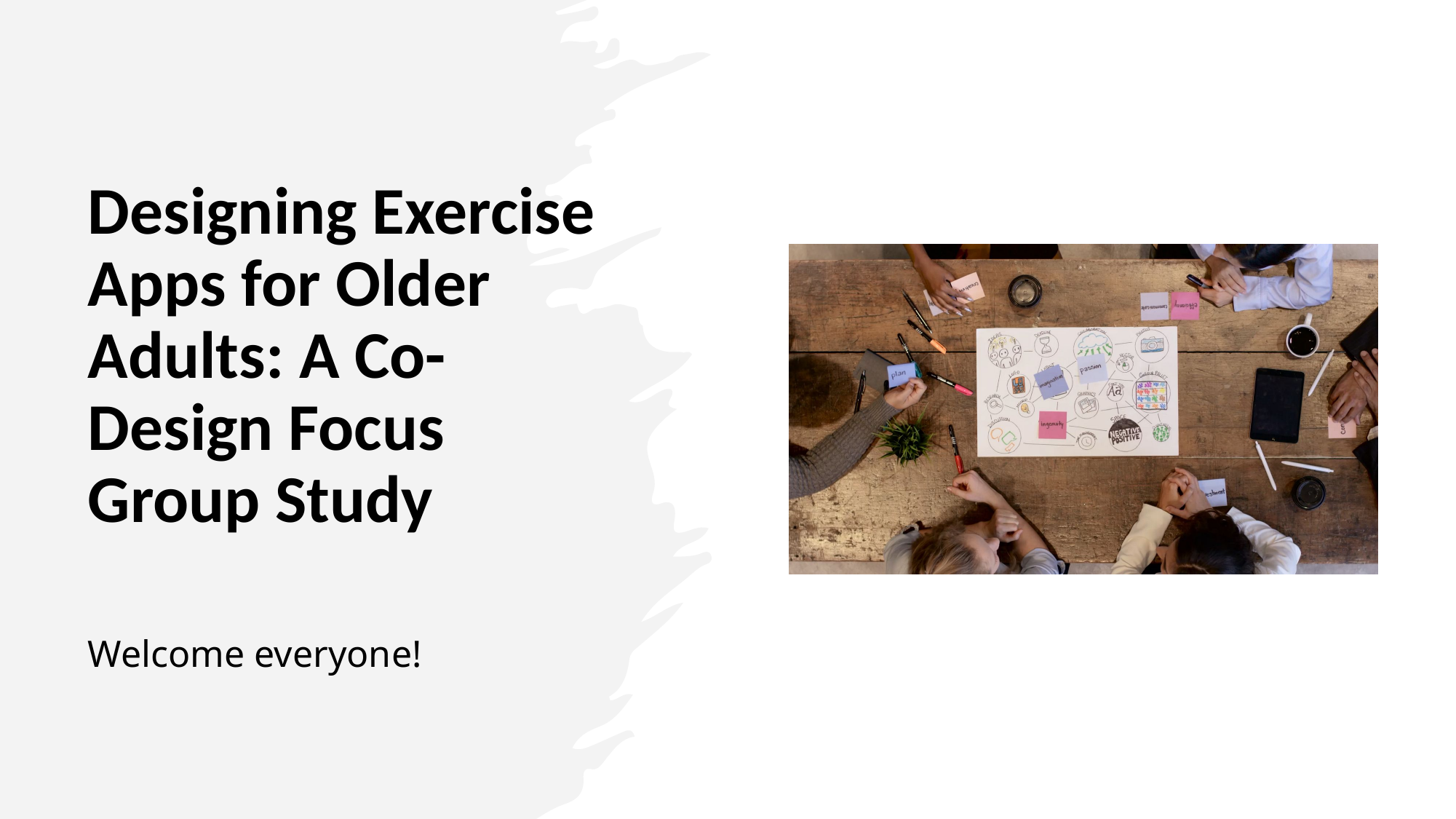

# Designing Exercise Apps for Older Adults: A Co-Design Focus Group Study
Welcome everyone!

## Slide 2
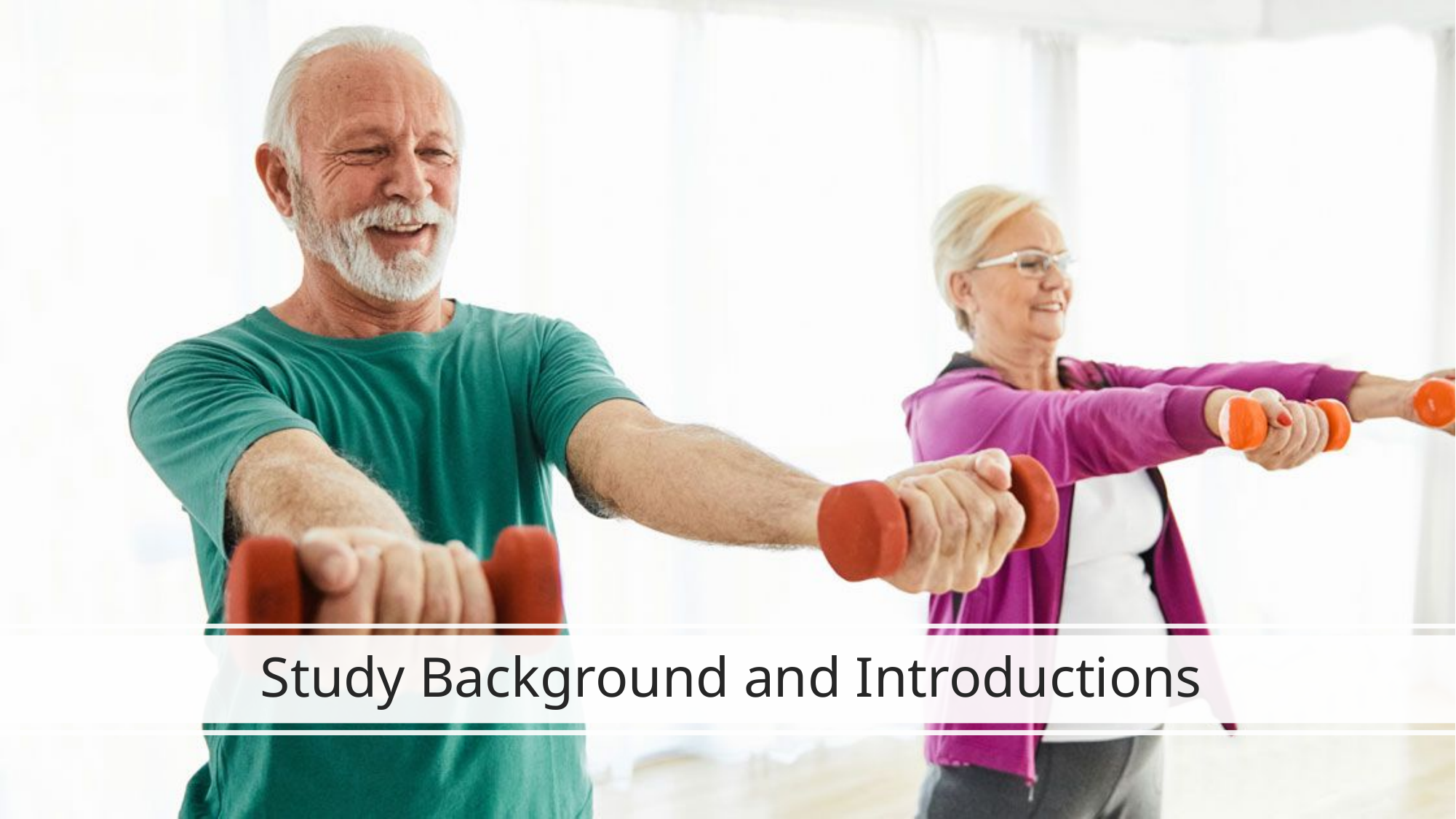

# Study Background and Introductions

## Slide 3
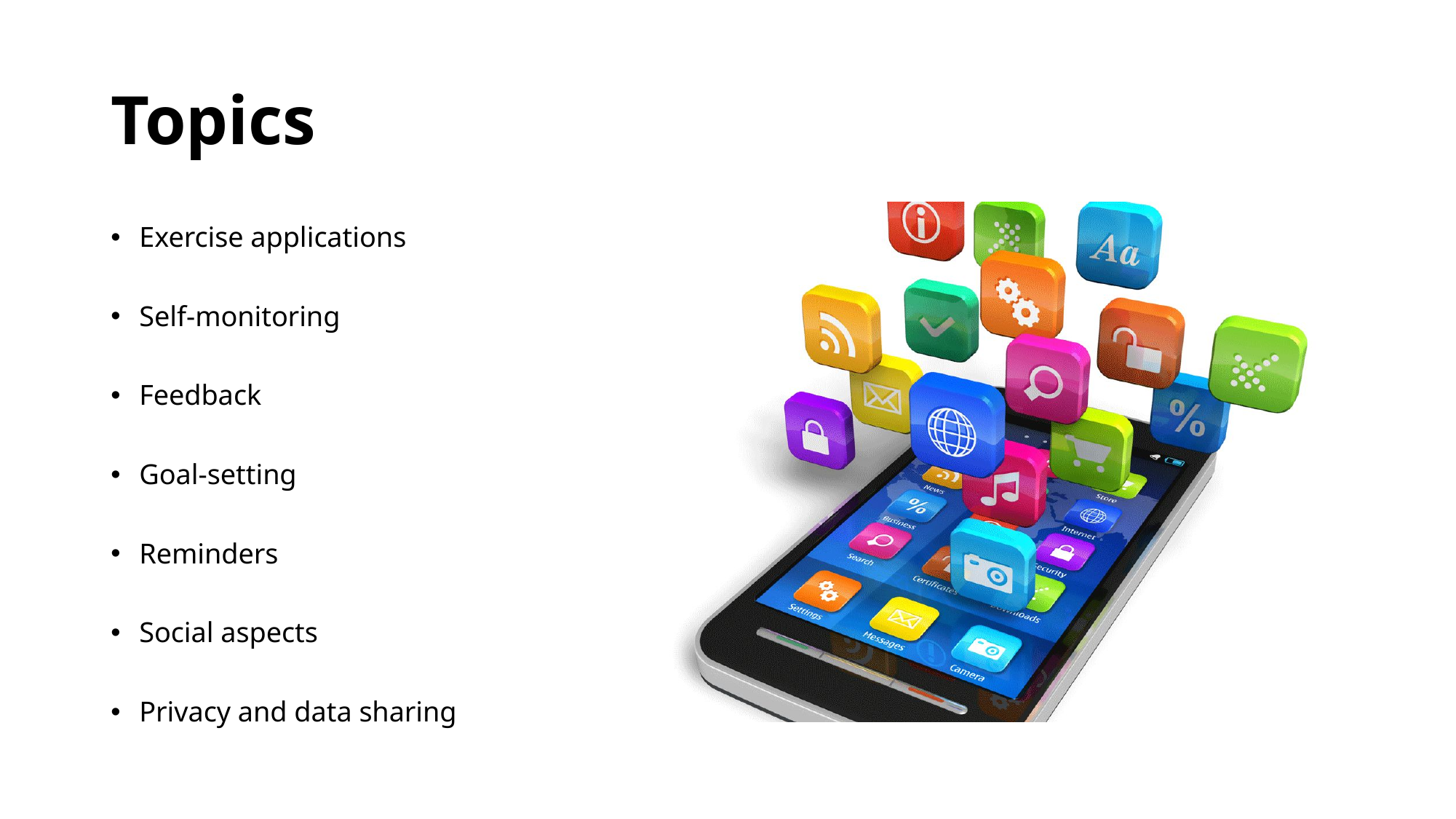

# Topics
Exercise applications
Self-monitoring
Feedback
Goal-setting
Reminders
Social aspects
Privacy and data sharing

## Slide 4
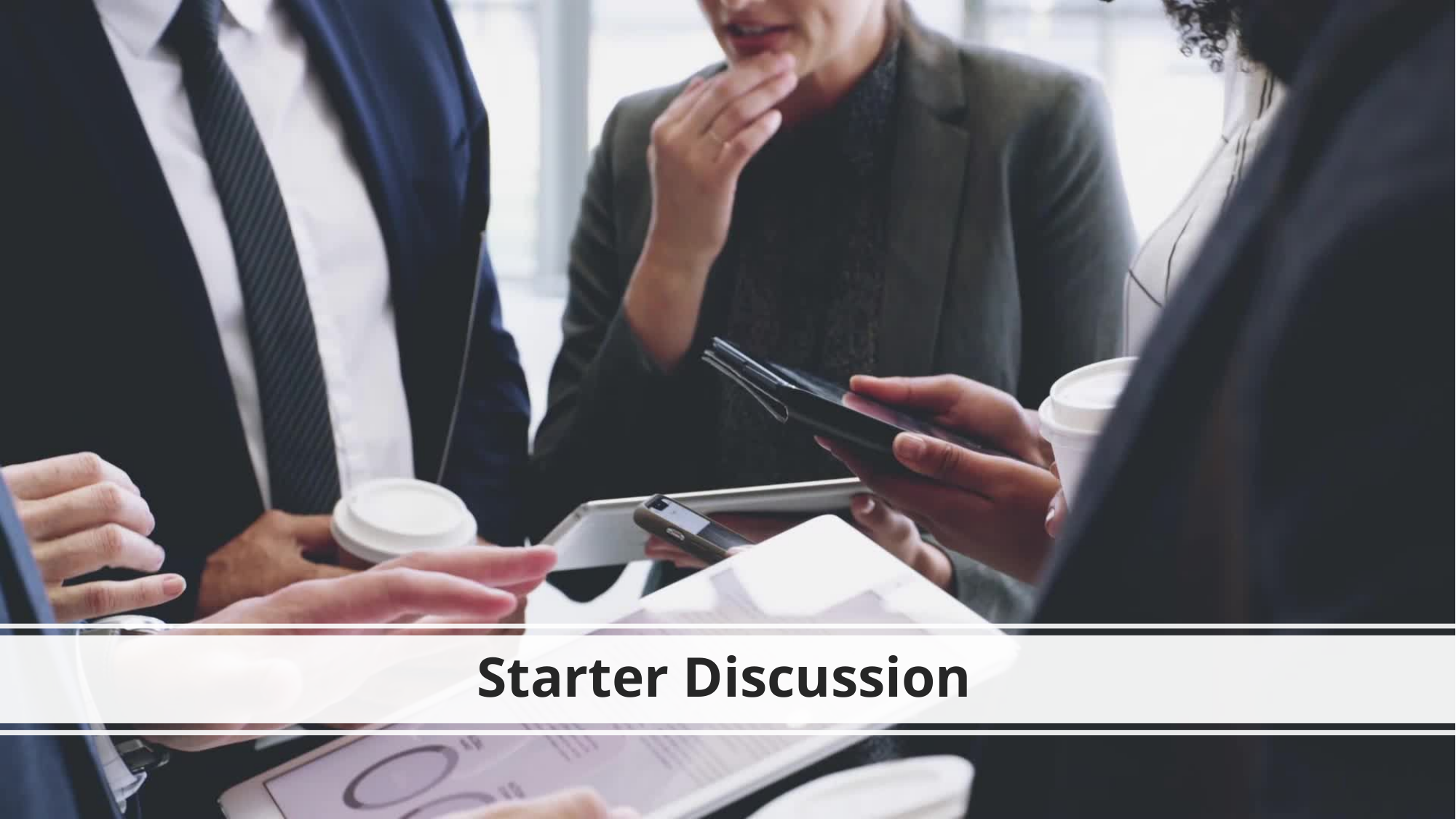

# Starter Discussion

## Slide 5
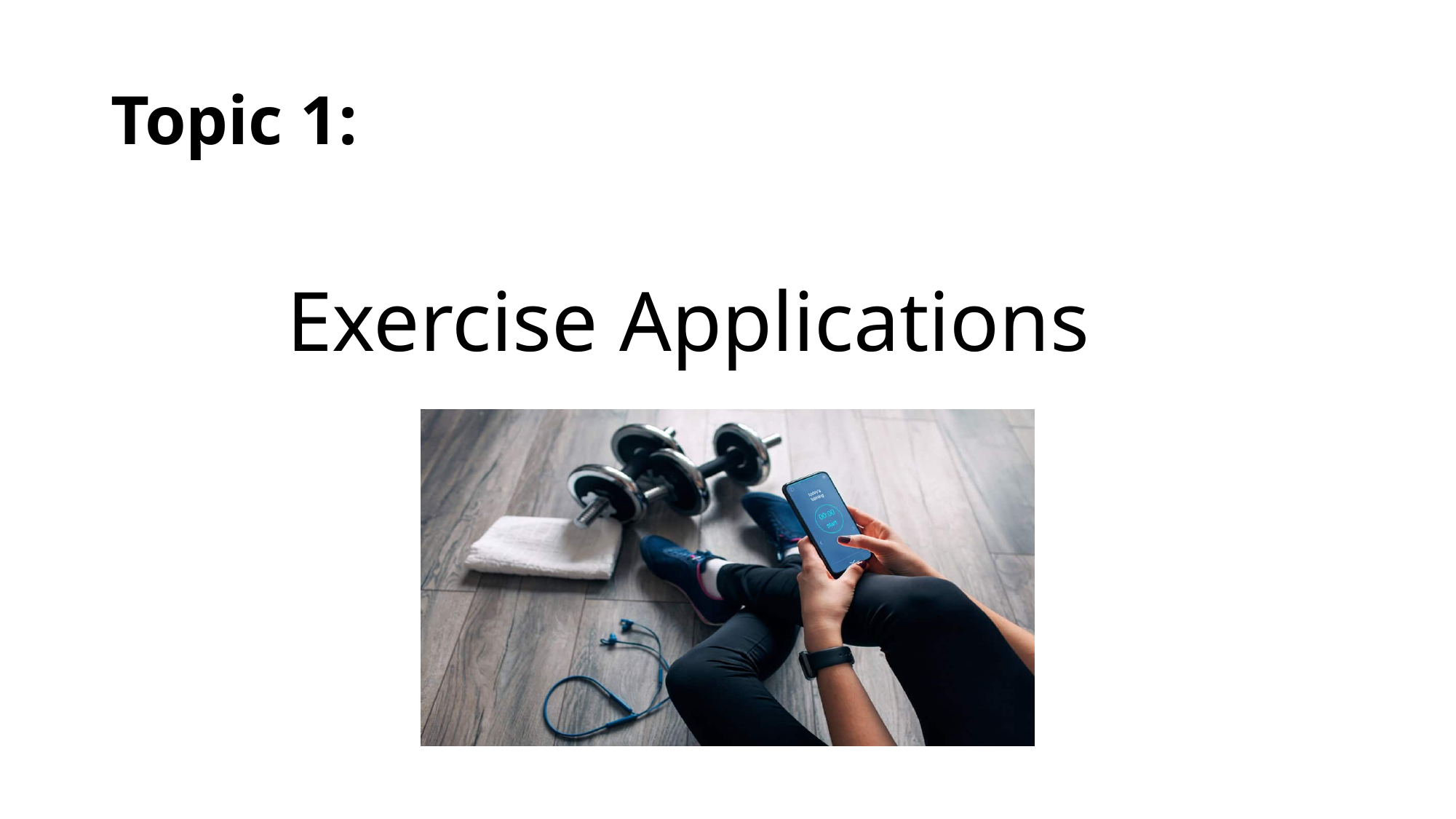

# Topic 1:
Exercise Applications

## Slide 6
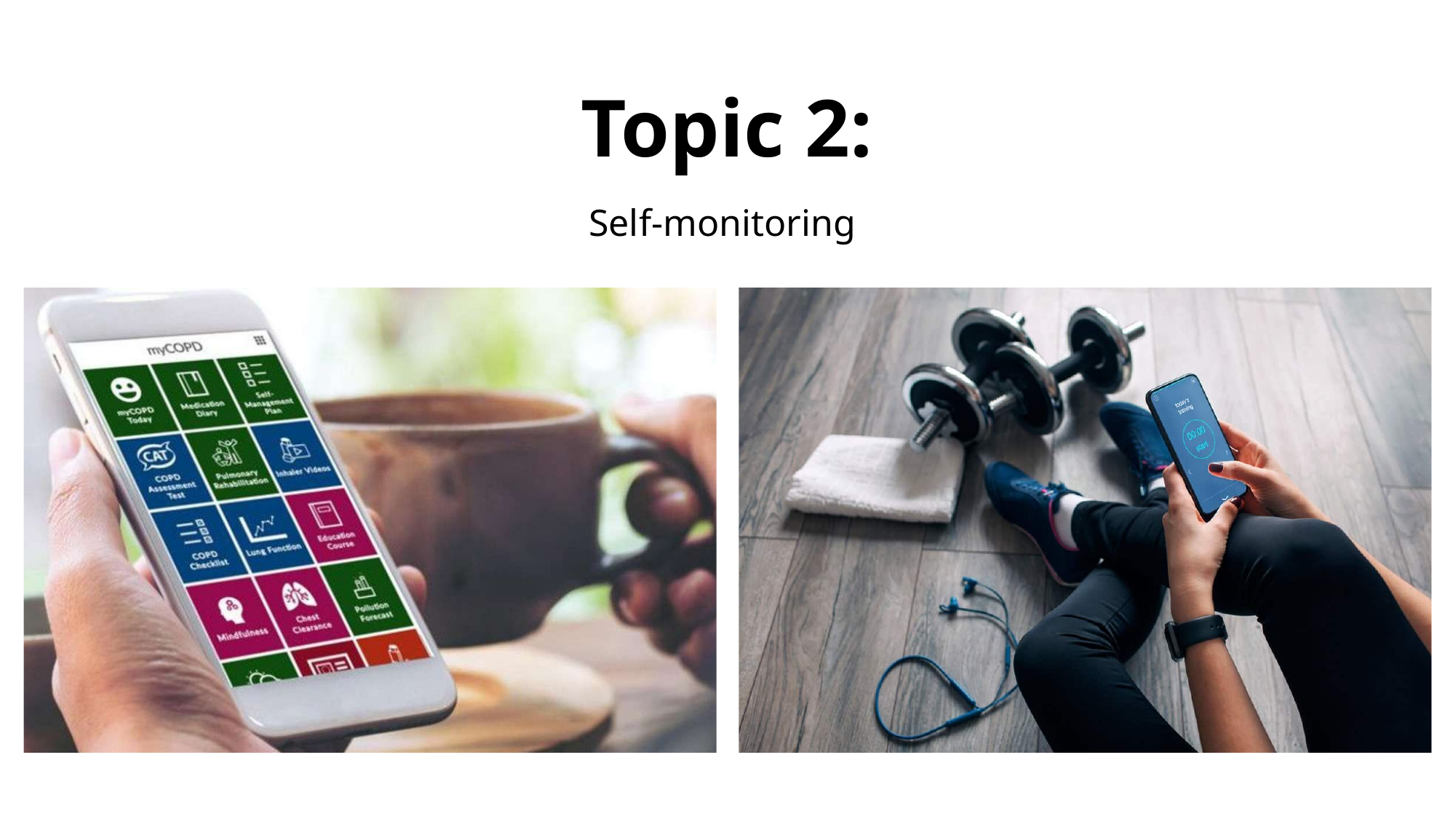

# Topic 2:
Self-monitoring

## Slide 7
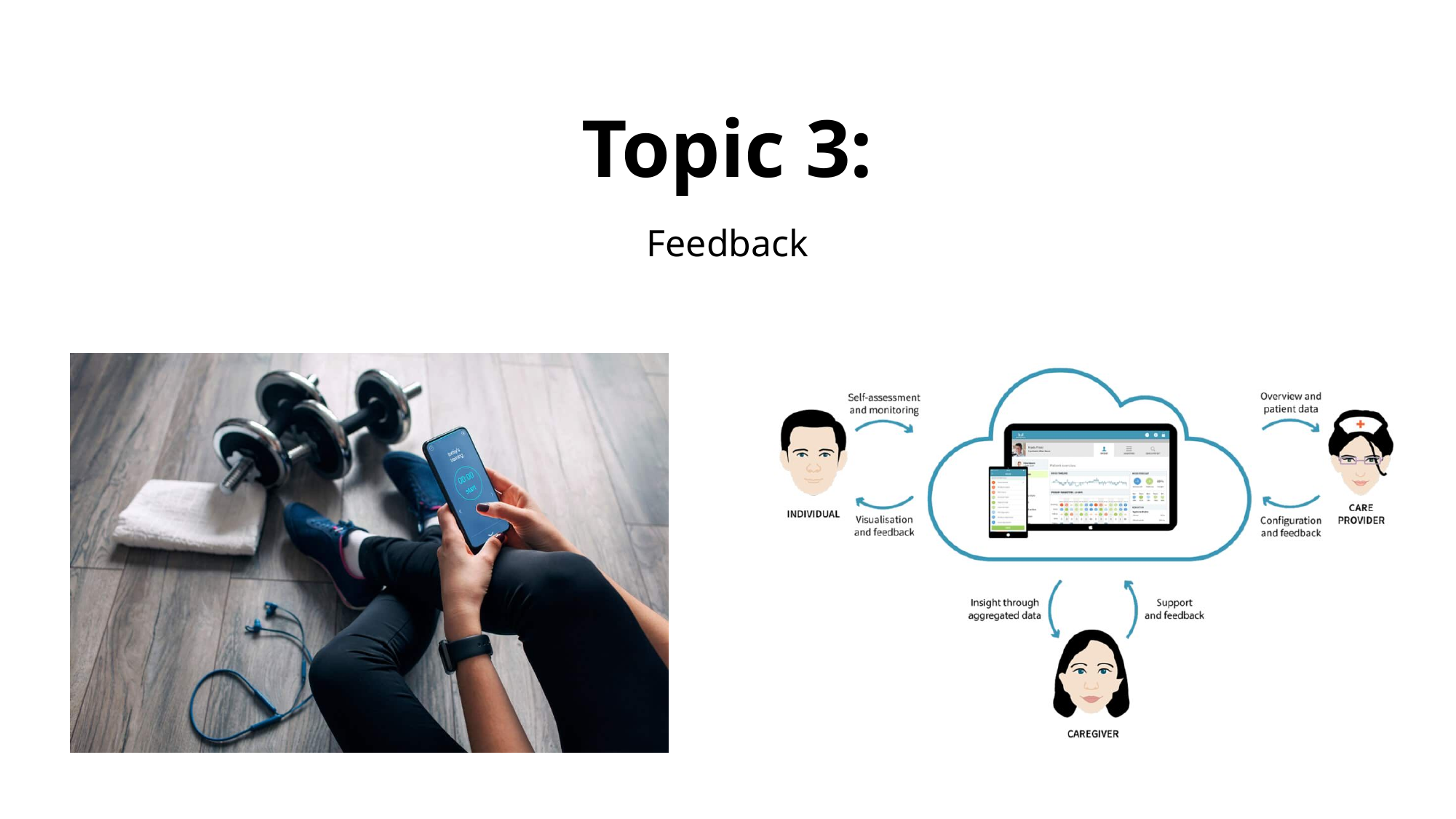

# Topic 3:
Feedback

## Slide 8
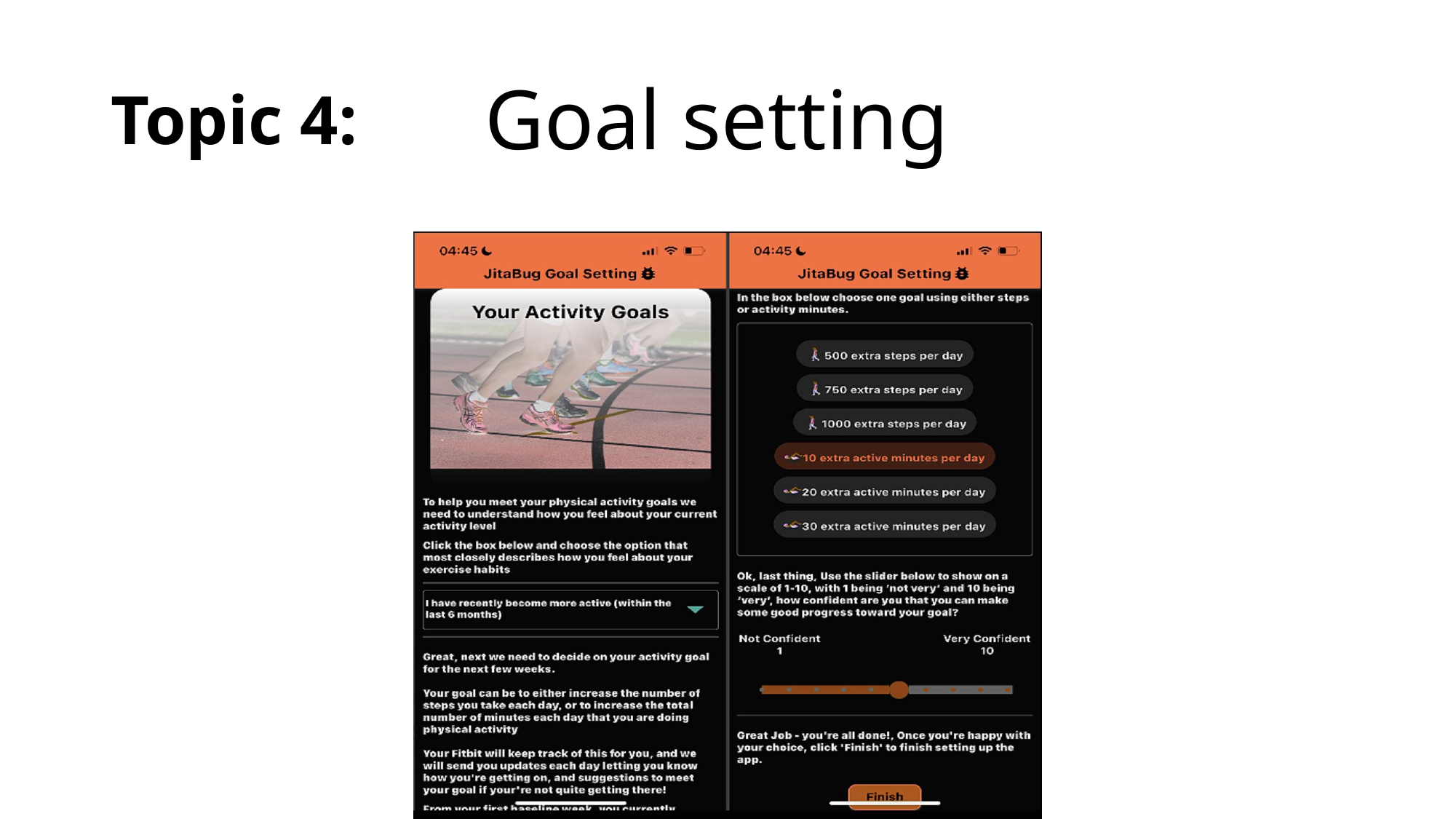

# Topic 4:
Goal setting

## Slide 9
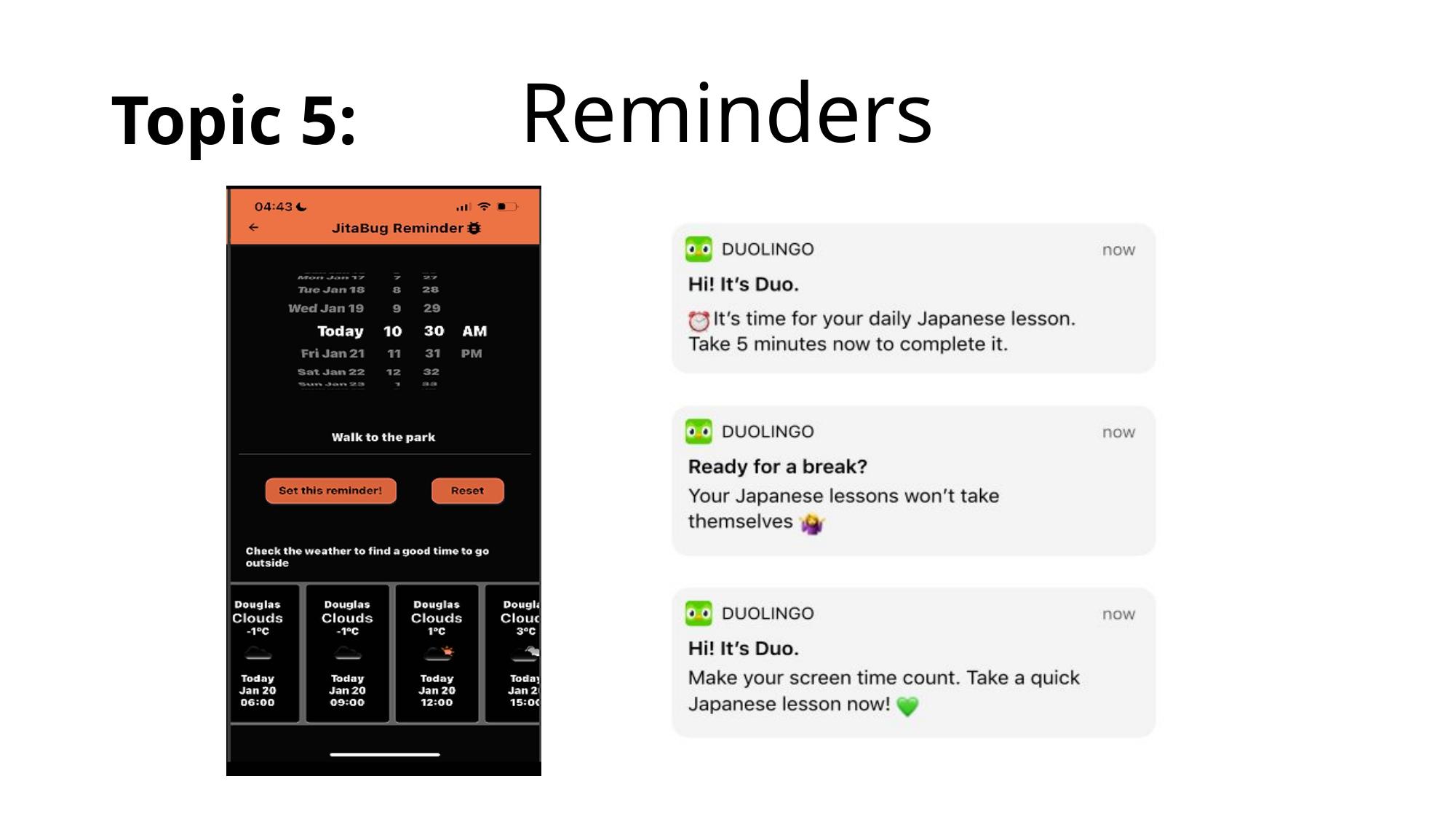

# Topic 5:
Reminders

## Slide 10
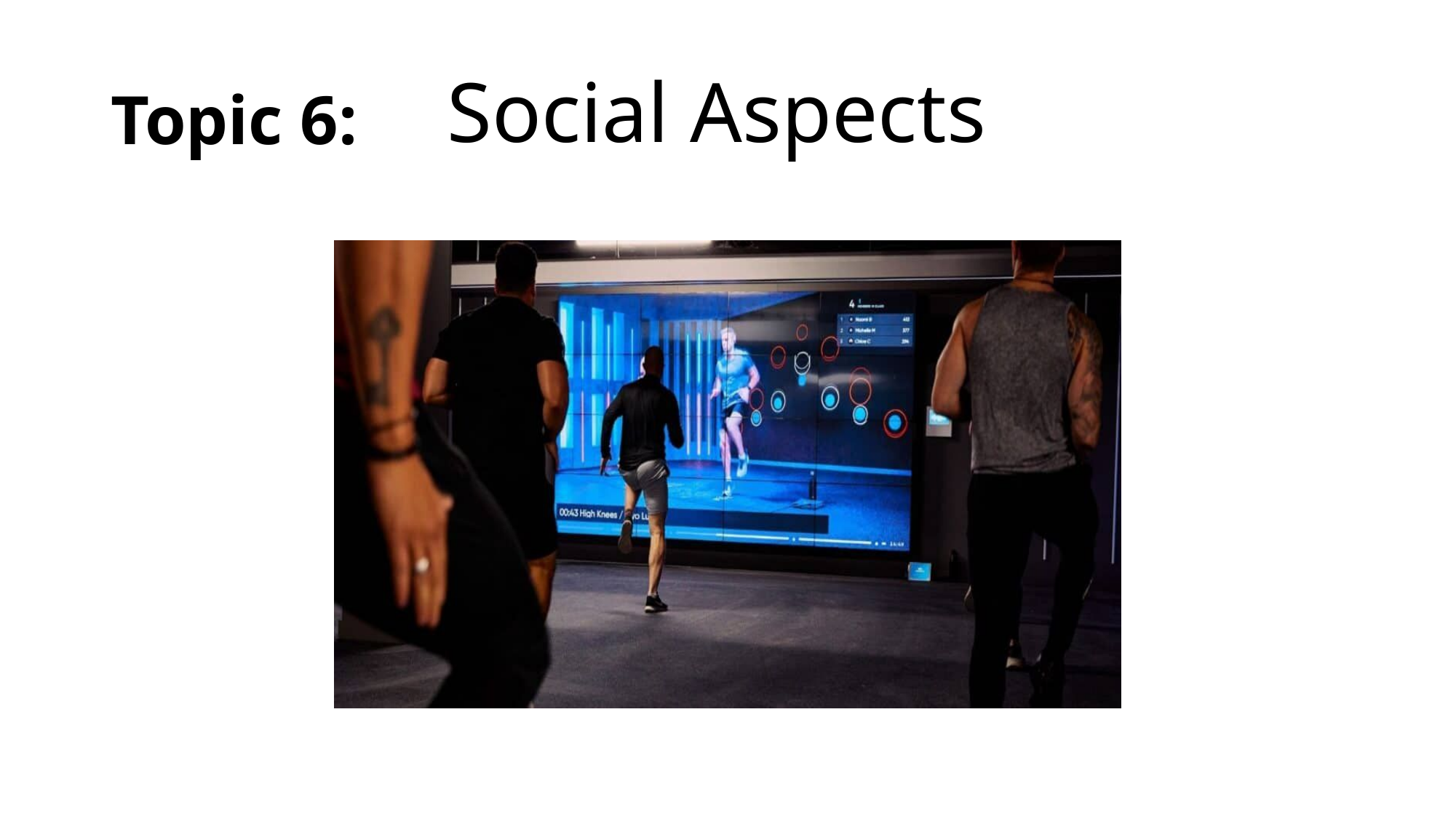

# Topic 6:
Social Aspects

## Slide 11
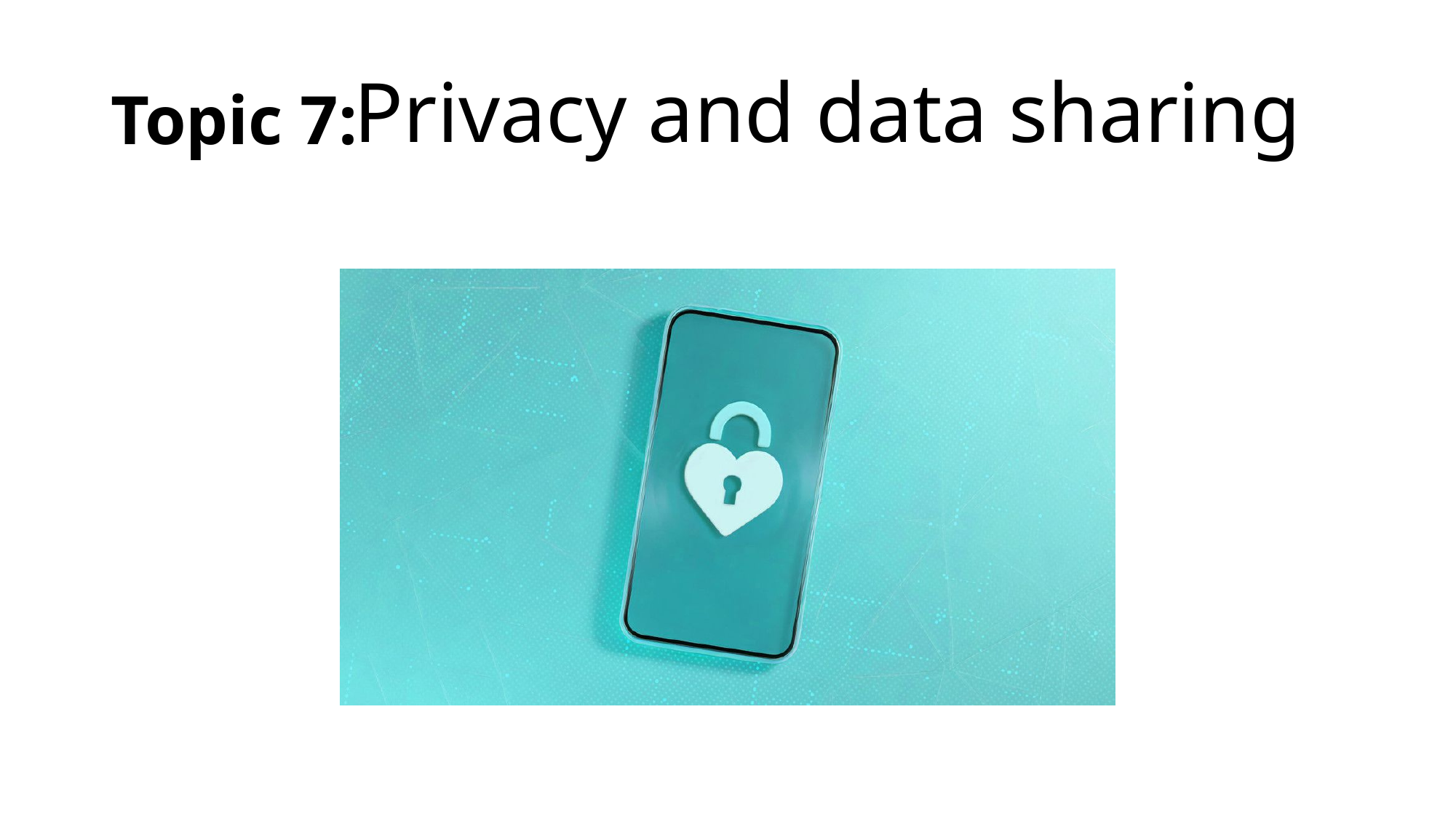

# Topic 7:
Privacy and data sharing
