# Supplementary material for: Designing an mHealth App to Encourage Uptake of Muscle-Strengthening Exercise in Older Adults: Co-Design Focus Group Study
Source: JMIR Aging. 2026 Mar 12;9:e87332. doi: 10.2196/87332 (PMC13022557; doi:10.2196/87332)
Supplement: Multimedia Appendix 4 [file aging_v9i1e87332_app4.docx]

**Quote Table**

| **Participant** | **Transcript Excerpt** |
| --- | --- |
| ***Theme 1: mHealth as a tool for supporting health and wellbeing*** | |
| *Participant 6 (experienced participant)* | ***‘****A part of my app. It tells you if you’ve been sat for a long time and it just basically says maybe it’s time to get up and move, which I think is quite good because sometimes you get engrossed in something and you think, yeah, OK, I’ve sat here for a bit of time.’* |
| *Participant 3 (experienced participant)* | ***‘****I use an app when I am swimming, which is built into my swim goggles. Find that very good, it’s called ‘Form’ it also has a heart rate monitor attached, which gives you a kind of indication of how hard or easy you’re exercising and also gives you the kind of distance you’re swimming and stroke rate, that kind of thing. It’s probably too much information, but it’s quite useful.* |
| *Participant 6 (experienced participant)* | **‘***Definitely have it featured on a watch or whatever as well as the app, because then you’re getting instant feedback. For instance, when I first started the exercise classes and it was different work out from being in a pool, my heart rate went really up, and I knew instantly. I got the feedback instantly, so I knew exactly what was going on in my body and I was pushing myself.’* |
| *Participant 2 (experienced participant)* | *‘I’ve noticed aches and pains, as I’ve got a bit older and would like to probably be stronger, definitely lost strength and so for me probably strength is one of the things that I would be keen to maintain as I get older.’* |
| *Participant 3 (experienced participant)* | ***‘****I’m starting to find lifting heavy things a bit of a challenge. So I’m wondering if I’m starting to lose a bit of muscle mass, although it’s not really affecting my other activities, but yeah, something that would encourage me to do some lifting. Lift weights would be really useful.’* |
| *Participant 17 (experienced participant)* | *‘About the past year, I’ve really tried to get more like strength exercises. You know, weight training and things because I’ve got osteoporosis, I had to have my hip replaced.’* |
| ***Theme 1 (subtheme): Overcoming barriers to accessibility*** | |
| *Participant 16 (experienced participant)* | *‘But it’s getting as you say, it’s getting involved in those kinds of things and taking that step and thinking that I’m not fit enough to go into this kind of thing or are they going to be looking for somebody that’s really gym fit.’* |
| *Participant 17 (experienced participant)* | *‘But I only ever went to classes and never very rarely used the gym because I didn’t really enjoy it at all. And I liked, you know, I’d go to class for an hour, but I wouldn’t really go to the gym for an hour. So like, recently the past year or so, it’s only because I went to the personal trainer, you know, obviously to help as well. Due to the app I use, I have a goal, and I’ve got a routine now, and as some of you know, a lot of times you don’t feel like it.’* |
| *Participant 18 (experienced participant)* | *‘So, it’s almost like it’s a catch 22. How do you get fit when you feel you’re not fit to get fit? maybe an app could help.’* |
| *Participant 8 (inexperienced participant)* | *‘I used to go to YouTube. I’ve discovered fabulous 50s. For people who are over 50 and then it’s just as a range of different exercises, you know, usually they’re just walking included in it, and it means you can do it in the house or whatever’* |
| *Participant 15 (inexperienced participant)* | *‘I just go onto YouTube and I’ve got a sports watch. I don’t use an app for a specific exercise. It’s just really YouTube which fits into my routine.’* |
| *Participant 1 (experienced participant)* | *‘Just another thought on that one. It would be good to have these with equipment that can be accessed in a domestic routine. So, you know, I’m not entirely sure what I mean by that. But rather than expecting people to have, you know, weight sets or whatever get, you know, used items that people have readily accessible round their houses.’* |
| *Participant 6 (experienced participant)* | *‘Yeah, that’s a good point, somebody said that as well that you know you can get a bottle sometimes, they’re kind of shaped thinner in the middle and then they go out to fill it with sand.’* |
| ***Theme 1 (subtheme): Potential for educational support via mHealth*** | |
| *Participant 6 (experienced participant)* | ***‘****I do use an app on my Huawei watch, and it does give you a lot of information that I basically didn’t know about myself which is quite motivating as well.’* |
| *Participant 7 (inexperienced participant)* | *‘So, an app that was maybe more suited towards what we need at our age would be useful and relatively simple but without all the extra things, just the things that you need to know to improve your muscle strength. Perhaps. So yeah, I would be interested in that.’* |
| *Participant 10 (inexperienced participant)* | *‘But what I also use it for is educational stuff. There’s some stuff I use. It could be anything from professional stuff too such as YouTube to show me how to stick a fence up or something in the garden, so it’s got educational uses as well.’* |
| *Participant 4 (experienced participant)* | *‘But just to echo some of the sort of sentiments that have come, I’m realising that while I’m fit, I’m not flexible. I’ve not got the sort of strength that I probably want to have and just want to sort of educate myself into what the next steps could be just to sort of bring myself on.’* |
| *Participant 8 (inexperienced participant)* | *‘I don’t really do a lot of weight training, although I do realise that it’s it is important or that I’m beginning to realise it’s important. So, I think that’s something I would start if I had an app.’* |
| *Participant 15 (experienced participant)* | *‘I enjoy swimming, and I practise yoga, and I do use a health app and it’s just getting some more information on the muscle strength. You know, it’s then easier ways to obviously strengthen your muscles, there’s always new stuff you can learn.’* |
| ***Theme 2: Motivation and engagement through app features*** | |
| *Participant 3 (experienced participant(* | ***‘****That’s what I like about it. I just realised I got a reminder today that I haven’t done my third run of the week, so that’s good. I’ll need to sort that out. So even that kind of thing’s quite helpful because I thought I had.’ (Participant 3, experienced participant)* |
| *Participant 4 (experienced participant)* | ***‘****I’ve got my Garmin app that that tells me to move if I’ve not done more than a set number of steps in an hour. It does prompt if you’ve not done your 10,000 steps in the day, but that’s a sort of motivational bit to check and see where you’re at and just sort of like hit that target. So, it’s a real basic one.’* |
| *Participant 15 (experienced participant)* | *‘I’ve got a sports watch. So, comes back to the app and the apps are in your life if you like it or not and you don’t realise how much they are actually. So, I do go into my apps to see how many steps I’ve done in a day and things like that. So yeah, I think an app, if you know, even an app or something on your computer if you’ve not got a sports watch. Yeah, I think it is quite beneficial because it shows you how you’re progressing. I think we do need to have that feedback, personally.’* |
| ***Theme 2 (subtheme): Progress tracking as a source of motivation*** | |
| *Participant 7 (inexperienced participant)* | *‘I think it’s motivating. I think you do have to have something that says you’re improving, so if you can see improvement, it’s definitely motivating, and it helps you to keep going.’* |
| *Participant 17 (experienced participant)* | *‘My gym app, it’s quite good. It’s good motivation as well, because sometimes if you’re doing exercise over, you know, a few months like say, six or eight months and you’re just gradually increasing your weight and things and you do forget what you said, you know, so looking back like four or five months ago, you think, oh, I’ve got a better since then, you know, because you can see your, you know, your progress.’* |
| *Participant 18 (experienced participant)* | *‘With regard to the apps I’ve used, I can’t even remember the name of the one that was measuring the steps, but at least you had a measure so you would know if you were doing 5000 or 6000 because you had a number there. Same with weight loss and you would have a number, a big number or a wee number.’* |
| *Participant 7 (inexperienced participant)* | *‘Things like repetition of various things. So, if you start off only being able to do maybe five of a specific strengthening exercise and by the end of a month you can do 10, you’ve obviously got to have monitored that you’ve increased from 5 to 10 and that’s that is helpful. I feel as well and as I said before, motivating. Yeah.’* |
| *Participant 9 (inexperienced participant)* | *‘I think as you say there, if you’re doing 5 and you’d be able to build up to 10 and over the months you’ve maybe got up to 20, then you can we can relate to that, that progress that you’ve made because it’s you know it’s there in front of you and it is a personal thing.’* |
| *Participant 8 (inexperienced participant)* | *‘Well, I think it’s as we’ve just discussed, if you if you’re going down the road and using the app, then you do want to know what your progressing in.’* |
| ***Theme 2 (subtheme): Rewards and streaks as reinforcement*** | |
| *Participant 12 (inexperienced participant)* | *‘And when I do Wordle every day and I got, I got fanatical about continuing my streak on Wordle and it’s up to 138 days. Then went on a cruise and I was at sea one day and we couldn’t get, I couldn’t go online so I lost my streak, and I was devastated. So, I think you know having that way of sort of keeping the continuity and keeping that motivation to do something every day is quite good if it’s done in the right way.’* |
| *Participant 18 (experienced participant)* | *‘Like it’s probably a wee bit of what you would call positive reinforcement. You know you’ve done 3 days, you’ve got, you know, well done, you’ve only got 2 to go, you know that’s massive.’* |
| *Participant 9 (inexperienced participant)* | ***‘****I personally think it’s extremely condescending if you’re going to give me anything, send me a box of chocolates you know, or give me a glass of wine. I do not want a star, my Lord. No, I don’t want a star or a streak.’* |
| *Participant 10 (inexperienced participant)* | *‘Yeah agreed, I’m not going to suggest a star, but I, but I would appreciate some feedback on you how I’m doing. I’m a boring guy. I like graphs, you know? Give me something to say this. A dotted line to say this is where we think you should be and then an actual line to this is where you are. I can do that. I accept wee celebratory things popping up and exploding in front of your eye. It’s a wee bit kind of old school and patronising, but I think you still need to find a way of saying. Well, are you are on track at least.’* |
| *Participant 9 (inexperienced participant)* | *‘Sorry, I do appreciate it because I think you have to build everything in for everybody. There may be people that you know will want a star there. But I mean my Fitbit gives me graphs and I’ll be honest, I don’t look at them. No, I think you need to build it so that it is all-encompassing. It’s not just for me. You know, as individuals. And so yeah, I think you are right. You do need some sort of understanding of where you are throughout the journey, however long or short or intermittent that may be.’* |
| ***Theme 3: Drawbacks of current mobile apps*** | |
| Participant 8 (in*experienced participant*) | *‘Yeah, I was reluctant to use a lot of apps initially I think, but I do use it for shopping and things as well, so yeah, text and WhatsApp and stuff, but that’s really about it.’* |
| *Participant 10 (inexperienced participant)* | *‘I’ve got a problem with sleep trackers from work perspective. They induce more anxiety than they help to be perfectly honest people start to worry that they’re not getting enough and then you can’t make yourself sleep.’* |
| Participant 9 (in*experienced participant*) | *‘I’m quite cynical about apps at the moment out there, because they don’t actually relate to my age group.’* |
| *Participant 7 (inexperienced participant)* | *‘So, an app that was maybe more suited towards what we need at our age would be useful and relatively simple but without all the extra things’* |
| *Participant 7 (inexperienced participant)* | *‘I did try to find something that was related to strength for older adults but there’s a lot of stuff out there. It’s quite hard to find what you need.’* |
| ***Theme 3 (subtheme): Complexity and poor usability*** | |
| *Participant 17 (experienced participant)* | *‘I’ve got like a Garmin watch, so I have tried that, but I don’t really like it. You know that’s a bit complicated. I have tried other things like over the years like Strava and things like that, but I don’t, I don’t like them. I think they are maybe you know complex for what I would be using it for, they’re too kind of advanced.’* |
| *Participant 8 (inexperienced participant)* | *‘I started using it and there’s all the things for tracking and you know, making sure you come back every day. But I have. I have lapsed a bit. I don’t really enjoy it. It wasn’t great. I don’t know why there’s a lot of stuff in it. Take like all these things that sometimes takes you ages to work your way around the app, and so it’s probably too complex to find, you know, just to do what I, what I want.’* |
| *Participant 18 (experienced participant)* | *‘I did use an app a long time ago and it was measured in steps, and you had to connect a watch. I can’t remember the name of it. I do have a watch. It’s not an apple or a Fitbit, but it’s one of those watches that you can set up to connect with the app, but I’ve not been able to set it up. So, you know I’ve been technologically minded, but the setup has actually been a bit of a barrier in me utilising that watch and app.’* |
| *Participant 3 (experienced participant)* | ***‘****So, I mean, just the simplicity of that, I suppose what I’m getting at, you know that, that to me is a really good thing. And I’ve seen some other apps that, you know require a level of understanding that probably most of us have, but you know, I don’t think they’re particularly user friendly for people who might be considering taking their first steps into exercise in later life.’* |
| *Participant 12 (inexperienced participant)* | *‘Yeah, something as easy and straightforward as possible. I mean, I, although I’m reasonably IT savvy, I would have thought, but you know it the easier it is. Less sort of features that are sort of unnecessary, I think would certainly help. From my point of view.’* |
| ***Theme 3 (subtheme): Scepticism over effectiveness, safety and evidence base*** | |
| *Participant 10 (inexperienced participant)* | *‘I manage occupational health at NHS Lanarkshire, and we see first-hand what muscle wastage etcetera does to the working population, so I have a professional interest as well. I’m a wee bit cynical towards some of the apps that’s out there already. It’s more just flattering to deceive, to be honest, but I think* ***Researcher*** *touched on it. There was very little on the impact of it all and that’s the world I’ll live in.’* |
| *Participant 10 (inexperienced participant)* | *‘More for me would be I’m quite interested in how you would, how your algorithms and your apps would work to inform me that I’m actually improving or I’m getting stronger etcetera.’* |
| *Participant 11 (inexperienced participant)* | *‘Because nobody in the app tells me you’re not doing it right, but if you’re in a live class, yes, you know, that’s what you do. I do a stretch for the kid’s football when I was coaching the kids. And do you know the kids could see me doing it and you could see them looking at me and then trying to do that. And I felt that was a big thing*. *So, with an app you would have to work out some kind of way of checking that you’re doing it correctly.’* |
| *Participant 17 (experienced participant)* | *‘I use a couple of videos on my app as we’re starting to talk about the videos, it’s like physiotherapists, really, because I think a lot of videos you go on and even now with my personal trainer because I went to him to make sure my technique was good so I didn’t injure myself. And now I look at a lot of videos and I think these people are promoting that and it’s dangerous things they are doing, you know, when they’re promoting it for older folk. And I think a lot of older people, do they have things like back issues, they have injuries, they have osteoporosis. They have a lot of things where they could be easily injured, and you see some of these online videos and you think that’s quite dangerous.’* |
| *Participant 5 (experienced participant)* | *‘Because I’m working on a strict programme at the minute to avoid surgery,  the feedback I’m looking for is specific, so it’s not that easily obtainable’* |
| *Participant 10 (inexperienced participant)* | *‘You’re in a kind of unique demographic. You’ll have tonnes of stuff on. You know, if you’re doing a cardiac workout, then this is the zone you should be burning in and all that sort of stuff. So, all well-known and that will transfer by age. But what other apps don’t do is appreciate well, that’s fine for the average 65-year-old, but what about the 65-year-old on anti-hypertensive and antarthritic drugs and all the rest of it.’* |
| ***Theme 4: Desired app elements and features*** | |
| *Participant 1 (experienced participant)* | *‘Simple navigation would be good. Definitely. Yeah, yeah.’* |
| *Participant 3 (experienced participant)* | *‘I’m just lacking motivation, but I find the couch to 5k app easy to use due to its simple interface.’* |
| *Participant 5 (experienced participant)* | ***‘****I think it’d have to high contrast colours just to be more visible and attract attention when I’m using it.’* |
| *Participant 10 (inexperienced participant)* | *‘Usability needs to be fundamental, and I think we’ve all probably been on websites. Whether you’re buying something on site or doing a bank transfer or whatever. Then some of them are better than others, you know, and most of them are kind of intuitively simplistic. So probably like those, not too busy and easy to follow. Is the kind of rule of thumb. You can debate around colours and all the rest of it until the cows come home. Really, it’s more around do the pages move correctly and does it take me where I want to go sort of thing?’* |
| *Participant 12 (inexperienced participant)* | *‘Yeah, something as easy and straightforward as possible. Less sort of features that are sort of unnecessary, I think would certainly help. From my point of view.’* |
| *Participant 15 (experienced participant)* | *‘Yeah, I do think larger text would be of benefit. You don’t want to be wearing glasses all the time. If you wear them, bigger text is beneficial.’* |
| *Participant 18 (experienced participant)* | *‘I think that large fonts are good a good idea and more and more I’m finding myself really and I’m wearing glasses now. But you know, trying to read small print.’* |
| ***Theme 4 (subtheme): Customisable reminders*** | |
| *Participant 9 (inexperienced participant)* | *‘Yeah, I think it’s a good idea if you’ve got like in the settings and you can turn on or off. If you can personalise the notifications that you get, I think that’s a really good idea.’* |
| *Participant 10 (inexperienced participant)* | *‘I like the idea of it being personable that you can pick it because I’m more of a day three. You know, if I’ve not done anything for a few days then give me a push. But you know, I’ll just look at it with distain. If it’s trying to tell me to do something tomorrow’* |
| *Participant 8 (inexperienced participant)* | *‘I think the morning for me, if that was the case.’* |
| *Participant 14 (inexperienced participant)* | *‘I think reminders in the morning too.’* |
| *Participant 18 (experienced participant)* | *‘For me, in the morning, you know, start of another week.’* |
| *Participant 7 (inexperienced participant)* | *‘I would say the afternoon, because if you’ve had a really busy day and everything has got away from you and you just haven’t thought about it like by the afternoon, it’s not too late. If it was the evening, it would be too late and you’d think Nah, that’s it.’ (Participant 7, inexperienced participant)* |
| ***Theme 4 (subtheme): Clear video demonstrations of muscle strengthening exercise*** | |
| *Participant 10 (inexperienced participant)* | ***‘****I like the idea. I don’t know if it was what Participant 9 meant. I like the idea of being able to slide back and forward. On the screen to get you to repeat. You know just easily rather having to reload the whole thing again to get to the bit you didn’t get, so things like that are quite useful’* |
| *Participant 17 (experienced participant)* | *‘On each bit, although he’s obviously showed me them before, but sometimes if the if it’s a new machine and you can click on there’s a wee word on it, you click on it, and it brings up a video of somebody sort of using the machine. It’s a lot easier.’* |
| *Participant 15 (experienced participant)* | ***‘****I’m quite a visual person, so I prefer to see visually and then obviously supplemented with, you know, maybe pointers in and reps or something like that. Just to follow.’* |
| *Participant 10 (inexperienced participant0* | *‘That I was going to say I quite like animation rather than some buff 25-year-olds or supermodel showed me how he’d do stuff. Maybe better would have a granddad or a grandmother doing it, but don’t make them buff. A bit of realism and it would be useful.’* |
| *Participant 9 (inexperienced participant)* | *‘I think you’re right. You can’t have like a six-pack guy standing in front of you or like, some supermodel. It needs to be relevant to the people that you’re trying to educate.’* |
| ***Theme 4 (subtheme): Customisable exercise options and feedback*** | |
| *Participant 9 (inexperienced participant0* | *‘The other thing I quite like is that you can put the exercises that you want together and like a tab for yourself so that you can go to those exercises rather than filing through 30 different types of exercises. So, you can move them into a tab that’s suitable for what you want to do, move them back, and then create a new programme for yourself.’* |
| *Participant 10 (inexperienced participant)* | *‘That’s a really good point, cause some people might just want to do leg strength and stuff and you know they might want to select bits and pieces, so that’s really relevant.’* |
| *Participant 12 (inexperienced participant0* | *‘I think participant 9’s idea earlier on to sort of kind of slide the various exercises into sort of your workout, your shortcuts or whatever you want to call it. And that could be changed as you as you progress. I think you know that gives you sort of one area within the app to go to get your sort of current work out regime.’* |
| *Participant 3 (experienced participant)* | ***‘****Maybe also you know some sort of element of customisation where everybody is started from a different point. That’s one of the things I don’t really like about classes at gyms. It gets kind of one-size-fits-all. So, if you’re coming into something like weight training, which I would certainly like to start, you know my cardiovascular fitness is probably OK. But as I said, I’m not brilliant. I’ve never really done much weight training. So, I mean maybe like you know, sort of staggered approach of beginners, intermediate advanced and tutorials based on each of those, and you can kind of find your own level rather than, I don’t know if you know that’s possible, but rather than the kind of one-size-fits-all approach.’* |
| *Participant 10 (inexperienced participant)* | *‘I don’t know if there’s a way to front load some questioning in the app so you can tailor it more to the individual, because you don’t want to set unexpected, you know, unachievable parameters.’* |
| *Participant 17 (experienced participant)* | *‘Yeah, I don’t. I don’t really like it because that might put you off as well, because if you’re working really hard and you’re like I’m still a beginner because I can’t get to this level, it would put you off maybe.’* |
| ***Theme 4 (subtheme): Preferences regarding social features*** | |
| *Participant 2 (experienced participant)* | ***‘****The option to do it as a as a class would be good because I’m not really motivated.’* |
| *Participant 4 (experienced participant)* | *‘I don’t actually like it because it’s seeming quite competitive if you get drawn into it. Maybe the public status of it? I know a lot of people have moved it more to private. You know, for their own personal view, rather than having it out there in the public.’ (* |
| *Participant 9 (inexperienced participant)* | *‘I think for lots of people, for me personally, it wouldn’t be either. There’s so many people talk online and don’t talk to people, they just talk. But for me personally you’ve got to remember you’re designing an app that’s going to integrate everyone. And I think that app there have the option to opt in or not. Then yeah. But I think there will be surprisingly a lot of people, 60 plus that will want to engage with a group on an app on their exercises.’* |
| *Participant 18 (experienced participant)* | *‘Yeah, I’m with Participant 17. I think if people want that option, then if it could be built into the app fine, but. You’re only doing it for yourself, aren’t you? It’s you. It’s your thing.’* |
